# Development and Validation of a Circumplex Measure of the Interpersonal Culture in Work Teams and Organizations

**DOI:** 10.3389/fpsyg.2019.00850

**Published:** 2019-05-01

**Authors:** Kenneth D. Locke

**Affiliations:** Department of Psychology and Communication Studies, University of Idaho, Moscow, ID, United States

**Keywords:** interpersonal circumplex, organizational culture, social norms, scale development, agency, communion

## Abstract

Interpersonal circumplex (IPC) inventories assess a range of dispositions but can condense and compare their findings within a circular model defined by two factors: agency and communion. Whereas other IPC inventories assess individuals, the current research introduces IPC inventories assessing the interpersonal culture (interaction and communication norms) characterizing an entire organization or team—namely, the Circumplex Culture Scan (CCS) and Circumplex Team Scan (CTS). Across an initial development sample (*n* = 1676), online validation sample (CCS, *n* = 808; CTS, *n* = 832), and onsite validation sample (CCS, *n* = 516 respondents from 21 organizations; CTS, *n* = 347 respondents from 38 teams), the eight 8-item CCS/CTS octant scales demonstrated good internal consistencies, circumplex properties, reliable within-group agreement and between-group variance (thus justifying aggregation across an organization/team), and convergent, discriminant, and incremental validity in relation to other measures. According to their members, the organizations/teams with the most satisfied members and customers/clients were organizations/teams with considerably stronger communal (e.g., being open and respectful) than uncommunal (e.g., being rude and guarded) norms and somewhat stronger agentic (e.g., being eager and assertive) than unagentic (e.g., being cautious and quiet) norms. The CCS/CTS complements existing IPC and organizational culture measures and helps bridge the IPC and organizational literatures.

## Introduction

A large majority of business executives believe that their organization’s culture affects their firm’s performance and overall value (Graham et al., 2019). Their beliefs are justified by an expanding literature demonstrating associations between measures of organizational culture and measures of organizational effectiveness (Sackmann, 2011), although the specific mechanisms through which culture shapes outcomes are just beginning to be delineated (e.g., Hartnell et al., 2019). The combination of what we know and do not know about organizational culture provides ample impetus for us to continue to refine our models and measures of organizational culture.

Although there are sundry definitions and measures of organizational culture, researchers concerned with empirical tractability and generalizability have increasingly focused on social norms as the essential core of the construct. To quote Chatman and O’Reilly (2016, p. 214): “…an appropriate starting point for a comprehensive theory of organizational culture is a focus on the norms that can act as a social control system in organizations. We believe this focus on cultural norms is appropriate both because norms translate into observable behaviors and attitudes, which are highly relevant for organizational psychologists and sociologists, and because informants can report on and articulate them.”

Norms are defined as “attitudes and behaviors that are shared by members of a particular group” (Haslam et al., 2011, p. 249). Two primary categories of norms are *descriptive norms* and *injunctive norms* (Cialdini and Trost, 1998). Descriptive norms are beliefs about how other group members tend to behave. Injunctive norms are beliefs about what behaviors other group members consider appropriate/desirable versus inappropriate/undesirable. Collectively, continuing compliance to perceived injunctive norms and conformity to perceived descriptive norms are the social processes that solidify and sustain the culture of an organization or team (Chatman and O’Reilly, 2016). However, currently, there exists no measure of organizational culture that specifically and comprehensively assesses the social norms for interpersonal behavior among organization members. The purpose of the current project was to develop such a measure and to thoroughly ground the measure on an established model of social behavior—namely, the interpersonal circumplex (IPC).

### Interpersonal Circumplex Inventories

Social motives and behaviors can be organized along two encompassing dimensions: Agency and Communion (for an overview, see Abele and Wojciszke, 2018). Agentic actions involve standing out and getting ahead—for example, by showing others your status, power, abilities, or accomplishments. Communal actions involve fitting in and getting along—for example, by showing others you are cooperative, kind, generous, or trustworthy.

Agentic and communal motives are human universals, and satisfying these motives is associated with better mental, physical, and social functioning (Locke, 2015). For example, people who effectively demonstrate both agency and communion are more apt to be invited into—and not be ousted from—cooperative partnerships, including work teams and organizations. On the other hand, agency and communion can entail costs. For example, pursuing agency can lead to wasting time and resources on fruitless ventures or competitions, and pursuing communion can lead to wasting time and resources giving to others who give nothing in return. In sum, there exist incentives to be unagentic (e.g., passive and yielding) as well as agentic (e.g., firm and assertive), and incentives to be uncommunal (e.g., wary and unsupportive) as well as communal (e.g., open and engaged). Perhaps for these reasons, people vary in their dispositions to be agentic, unagentic, communal, or uncommunal (Saucier et al., 2014). The current research proposes that dispositions to be agentic, unagentic, communal, or uncommunal can also characterize the normative interaction patterns among individuals within workplace teams or organizations.

**FIGURE 1:**
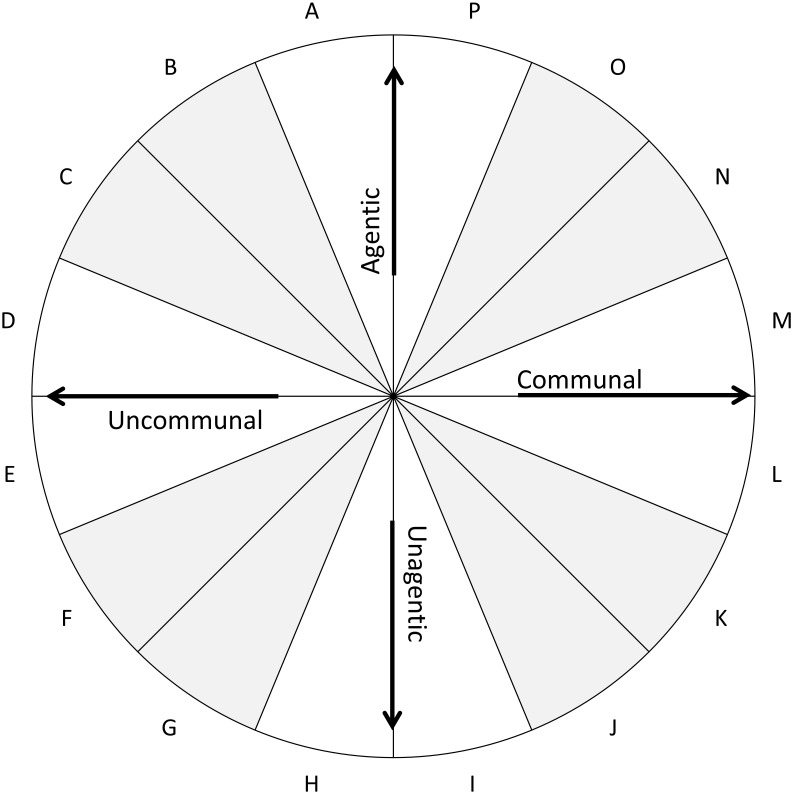
The interpersonal circumplex. The space can be divided into the following octants: PA, agentic; BC, agentic + uncommunal; DE, uncommunal; FG, unagentic + uncommunal; HI, unagentic; JK, unagentic + communal; LM, communal; and NO, agentic + communal.

As Figure 1 shows, the orthogonal dimensions of agency and communion define the IPC, a popular model for conceptualizing and assessing social perceptions, motives, and behaviors (Leary, 1957; Gurtman, 2018). The vertical agentic axis ranges from active, assertive stances (at the top) to passive, timid stances (at the bottom). The horizontal communal axis ranges from warm, affiliative stances (on the right) to cool, hostile stances (on the left). As one circumnavigates the circle, each segment reflects a progressive, weighted blend of the two axial dimensions; thus, adjacent segments are more similar than non-adjacent segments, with opposite interpersonal stances occupying antipodal segments.

The IPC has served as the foundational framework for measures of various constructs (Locke, 2011), including interpersonal traits (Wiggins et al., 1988; Markey and Markey, 2009), interpersonal problems (Horowitz et al., 2000; Boudreaux et al., 2018), interpersonal efficacies and strengths (Locke and Sadler, 2007; Hatcher and Rogers, 2009), interpersonal sensitivities (Hopwood et al., 2011), and interpersonal and intergroup values and goals (Locke, 2000; Locke, 2014), among others. Given their different foci, different IPC inventories may refer to the same IPC regions by different names; however, each 16th is also labeled with a letter code—shown in Figure 1—that is consistent across every IPC inventory.

There are several reasons for the growing interest in developing and using IPC inventories. First, as described above, IPC inventories are based on a theoretical framework that has proven relatively robust and generative across diverse research paradigms and areas of application (Locke, 2015; Abele and Wojciszke, 2018). Second, as will be explained and illustrated in Study 2, an individual’s or group’s scores across the different IPC segments can be condensed into a few trigonometric parameters, yielding assessment results that are both comprehensive and parsimonious. Third, because all IPC inventories share the same structure, administering different IPC inventories to the same individuals—referred to as *multisurface interpersonal assessment* (e.g., Cain et al., 2017)—can reveal informative consistencies or inconsistencies within those individuals across different types of interpersonal phenomena. For example, research assessing both interpersonal values and interpersonal problems suggests that people who place high value on avoiding humiliation are vulnerable to problems involving lack of agency (e.g., never speaking in group meetings; Locke et al., 2017).

### Bringing the Interpersonal Circumplex to the Workplace

Interpersonal circumplex inventories have been more widely adopted by clinical/personality researchers and practitioners than by organizational researchers and practitioners. Indeed, to my knowledge, the only IPC inventory specifically designed for organizational or workplace settings is the Circumplex Leadership Scan (CLS; Redeker et al., 2014), which assesses leadership styles associated with each IPC octant. However, the greater popularity of IPC inventories within the clinical field than the organizational field appears to be a chance outgrowth of the network of individuals and institutions within which the IPC was developed and disseminated. Accordingly, organizational assessment and intervention may benefit from the availability of multiple, complementary IPC instruments in the same way that clinical assessment and intervention have. Therefore, the aim of the current project was to develop an IPC measure that structurally mirrors the CLS, but measures a complementary construct—specifically, the interpersonal culture of a workplace team or organization. To highlight how it aligns with CLS, I will refer to this new inventory as the Circumplex Culture Scan (CCS).

Just as other circumplex inventories measure only those interpersonal dispositions modeled by the IPC (not an individual’s entire personality), the CCS measures only those interpersonal norms modeled by the IPC (not an organization’s entire culture). Culture is a capacious construct encompassing all the norms governing how members of an organization or team *work*; the CCS measures only that subset of norms governing how members *work together*. Thus, to be clear, the CCS does not seek to replace existing organizational culture measures (Jung et al., 2009) that measure other types of norms (e.g., relating to interactions with outside entities); rather, it seeks to complement them by providing more precise, comprehensive, and detailed coverage of the social norms guiding how individuals within an organization interact and communicate with each other.

The social norms assessed by the CCS may be of interest to organizational researchers and practitioners for several reasons. First, to the degree that the CCS measures norms not measured by existing organizational culture instruments, it may predict unique variance in organizational outcomes such as employee engagement and performance. Second, how members interact and communicate (e.g., norms governing sharing versus withholding ideas or concerns) can be a pivotal factor in the success of efforts to improve other aspects of organizational functioning (e.g., implementing new procedures or strategies). Third and most broadly, the CCS can provide a convenient tool for applying to organizational research and practice the IPC and agency/communion literatures’ empirical findings, theoretical models, and analytic techniques.

### Overview of Current Research

To develop and evaluate the CCS, I conducted a series of three studies. While the CCS is designed to assess either descriptive norms (what we do) or injunctive norms (what we should do), the current studies focused on descriptive norms. Study 1 employed an initial development sample to select items for the CCS scales. Study 2 used a separate, validation sample to evaluate the scales’ reliabilities, circumplex properties, and convergent, discriminant, and incremental validity. Finally, Study 3 evaluated the properties of the CCS in applied naturalistic settings, which afforded an evaluation of the effectiveness of the CCS in distinguishing among different teams and organizations.^1^

## Study 1—Development

### Participants

I used Amazon’s Mechanical Turk (MTurk) to recruit participants who were employed in the United States or Canada in an organization that had at least five employees. I collected two successive non-overlapping samples, both of which completed an online questionnaire. The survey software (used in this study and the other studies reported below) required respondents to respond to every item except the demographic items. For Sample 1, I only used responses from the 84.7% of respondents who did not give the same answer to >95% of the items, devoted on average ≥1 s per question, and whose responses to five repeated items did not deviate by >1 scale point on average. The resulting sample included 1,219 respondents (52.3% female; 77.2% White, 8.3% Black, 5.3% Asian, 9.3% other; *M* age = 37.0, *SD* = 11.3). For Sample 2, I only used the 82.6% of respondents who did not give the same answer to every item and whose responses to two validity-check questions deviated by <1.5 scale points on average from the correct responses. The resulting sample included 457 respondents (55.4% female; 76.8% White, 9.2% Black, 6.6% Asian, 4.6% other; *M* age = 37.3, *SD* = 11.7). The research reported in this manuscript was approved (or deemed exempt from review) by the University of Idaho Institutional Review Board and conducted in compliance with applicable ethical standards (including obtaining participants’ informed consent).

### Procedure and Results

First, with guidance from organizational specialists, I generated a list of 204 workplace social norms (e.g., “be honest with each other,” “criticize each other”). The items were designed to reflect the types of behaviors encompassed by the IPC as well as to be clear, succinct, and applicable to any work team or organization. The aim was to describe norms that diverse individuals—regardless of age, gender, cultural background, and so on—would readily recognize; nonetheless, individuals with different personal and professional experiences would likely have generated different items.

Second, Sample 1 participants indicated whether each item “describes how members of your organization interact with each other.” Each item was preceded by the stem “In this organization, people tend to” and followed by the following five-point (0-to-4) response scale: Strongly Disagree, Disagree, Neutral/Neither, Agree, Strongly Agree. Third, using their responses, I selected items using the classical test theory procedures used to create other IPC inventories (including the inventories cited in the section “Introduction”).

Specifically, I subjected the items to principal components analyses. Principal components analysis (rather than factor analysis) is used when developing and testing circumplex inventories because (a) the specific aim is to produce octant scales from which two orthogonal principal components can be computed as weighted sums and (b) the components (e.g., agency and communion) are treated as descriptive summaries of octant scores rather than as latent constructs *causing* octant scores, as assumed by factor analytic approaches. I identified which items were located within each 16th of the two-dimensional space defined by the first two components. Within each 16th, I retained items that showed strong correlations with the other items in that segment and overall communalities with the two principal components. This procedure culled the initial set of 204 items down to a smaller set of 80 items. A principal components analysis of these 80 items confirmed that two components—readily identified with the dimensions of communion and agency—explained most of the variance. Although these 80 potential items were somewhat evenly spread across every segment of the space defined by the two components, they provided relatively sparse coverage of the high-agency and low-agency IPC regions.

Therefore, I generated 24 additional potential items that exemplified either agentic norms (e.g., “strive to stand out”) or unagentic norms (e.g., “avoid standing out”) and that had not been included in the initial list of 204 norms. I administered the revised set of 104 items to Sample 2 and subjected the responses to a principal components analysis. Within each 16th of the resulting two-dimensional space, I retained the four items that demonstrated the strongest communalities and item-scale correlations. These 64 selected items constitute the CCS. Table 1 shows for each 16th an example item along with a summary name that captures the theme of the four items within that segment. To enhance parsimony and reliability, contemporary IPC inventories divide the IPC space into 8ths rather than 16ths (i.e., the differently shaded octants in Figure 1). Accordingly, the analyses below were conducted on the resulting 8-item octant scales (e.g., PA and BC).

**Table 1:** Circumplex Culture Scan (CCS) 16th scale names and example items.

Code	Name	Example Item: *Within my organization, people tend to…*
A	Pushy	Be eager to distinguish themselves
B	Competitive	Compete for status and prestige
C	Combative	Vie for power and control
D	Rude	Ridicule each other
E	Guarded	Avoid interacting with each other
F	Evasive	Avoid sharing their ideas with each other
G	Hesitant	Deny having ambitions to get ahead
H	Timid	Prefer to be followers than leaders
I	Cautious	Avoid saying anything controversial
J	Yielding	Avoid competing with each other
K	Modest	Be humble about their own successes
L	Respectful	Make each other feel valued
M	Open	Socialize with each other
N	Engaged	Offer each other constructive criticism
O	Confident	Express themselves decisively
P	Courageous	Be comfortable expressing unpopular opinions

*CCS, Circumplex Culture Scan.*

Table 2 reports the CCS octant scales’ descriptive statistics and reliabilities. (All data analyzed in the current paper are publicly available at this project’s Open Science Framework webpage^2^). All internal consistencies were adequate (ranging from 0.71 to 0.92) but were greater for scales at or near the poles of the communal axis (anchored by the LM and DE octants) than at the poles of the agentic axis (anchored by the HI and PA octants). Standard deviations also tended to be greater for the communal/uncommunal than the agentic/unagentic scales. Finally, respondents tended to describe their organizational norms as more communal than uncommunal and, to a lesser degree, more agentic than unagentic (e.g., LM and NO octant means were above the scale midpoint, whereas DE and FG octant means were below the midpoint).

**Table 2:** CCS scales’ descriptive statistics, intercorrelations, and loadings on communal and agentic principal components—Study 1.

				Correlations								Loadings	
Octant	α	*M*	*SD*	PA	BC	DE	FG	HI	JK	LM	NO	Communal	Agentic
PA	0.78	2.44	0.60	–								0.06	0.84
BC	0.87	1.97	0.79	0.52	–							−0.63	0.57
DE	0.92	1.21	0.85	0.00	0.59	–						−0.93	0.00
FG	0.83	1.29	0.64	−0.47	0.12	0.58	–					−0.62	−0.64
HI	0.71	1.70	0.58	−0.46	−0.27	0.03	0.56	–				0.02	−0.80
JK	0.86	2.10	0.76	−0.33	−0.65	−0.54	0.00	0.55	–			0.67	−0.60
LM	0.91	2.67	0.76	0.10	−0.47	−0.82	−0.49	0.07	0.62	–		0.92	0.01
NO	0.85	2.65	0.63	0.56	−0.02	−0.55	−0.68	−0.32	0.14	0.64	–	0.67	0.58

*N = 457. PA, courageous and pushy; BC, competitive and combative; DE, rude and guarded; FG, evasive and hesitant; HI, timid and cautious; JK, yielding and modest; LM, respectful and open; NO, engaged and confident. α, Cronbach’s alpha. Ratings were on Strongly Disagree (0) to Strongly Agree (4) scales. Correlations > 0.12 are significant at p < 0.01. The factor loadings reflect Procrustean rotation aligning the first two principal components with the theoretical orientations of the communal and agentic dimensions.*

Table 2 also reports the intercorrelations among the octant scales. Conducting a principal components analysis on these intercorrelations, the first two components explained 77.1% of the variance (communal axis: 42.4%; agentic axis: 34.7%). The octants’ loadings on these principal components revealed the expected sinusoidal pattern (see Table 2, rightmost columns): On the communal dimension, LM and (to a lesser degree) adjacent octants had positive loadings, whereas DE and (to a lesser degree) adjacent octants had negative loadings; on the agentic dimension, PA and (to a lesser degree) adjacent octants had positive loadings, whereas HI and (to a lesser degree) adjacent octants had negative loadings.

To the degree that the CCS forms a circumplex, the intercorrelations among its octant scales should meet certain criteria (Guttman, 1954). The minimum criterion is that correlations between adjacent octants circle exceed correlations between orthogonal octants, which in turn exceed those between octants 135° apart, which in turn exceed those of opposite octants. In total, a circular model makes 288 predictions about the relative magnitudes of correlations among eight octant scales. I tested if the CCS met these *inequality criteria* using a randomization test of hypothesized order relations (Tracey, 2000) as implemented by the RANDALL package for R (Tracey, 2016). RANDALL computes a Correspondence Index (*CI*) equal to the proportion of predictions met minus the proportion violated. The *CI* can range from −1.0 (all predictions violated) to 1.0 (perfect fit). RANDALL tests the *CI*’s significance against a null hypothesis distribution. In the current data, 283 out of 288 predictions were met, *CI* = 0.97, *p* < 0.0005, indicating excellent conformity to a circular model.

A more stringent criterion is that the observed correlations fit the ideal sinusoidal pattern of correlations expected if all eight scales (1) had equal communalities and (2) were equally spaced apart along the circumference of a circle (Fabrigar et al., 1997). I tested if the CCS met these *equality criteria* using confirmatory circumplex structural analysis/modeling (Browne, 1992) as implemented by the R package CircE (Grassi et al., 2010). When fitting restrictive circumplex measurement models, a root mean square error of approximation (*RMSEA*) ≤ 0.13 and comparative fit index (*CFI*) ≥ 0.90 are considered to indicate adequate fit (Gurtman and Pincus, 2003). The current data showed acceptable fit to the equal radius model (*RMSEA* = 0.13, 90% *confidence interval* = [0.11, 0.13], *CFI* = 0.95), the equal spacing model (*RMSEA* = 0.12 [0.10, 0.14], *CFI* = 0.96), and the model assuming both equal radius and equal spacing (*RMSEA* = 0.12 [0.10, 0.13], *CFI* = 0.95). In sum, the chosen set of 64 items showed good psychometric and circumplex properties.

## Study 2—Validation

Study 2 had four aims. The first aim was to evaluate the psychometric and circumplex properties of the 64-item CCS developed and refined in Study 1 in a new sample. The second aim was to explore if the CCS could be used to assess the culture of work teams as well as organizations. To distinguish these two targets, when assessing teams, I will refer to the inventory as the Circumplex Team Scan (CTS). The third aim was to examine the convergent and criterion validity of the CCS and CTS by administering additional measures of group culture and performance. The final aim was to explicate and demonstrate some of the special mathematical properties of circumplex inventories that facilitate summarizing, visualizing, and comparing assessment results.

### Methods

#### Participants

I used two survey panel providers (Centiment and TurkPrime) to recruit participants who were full-time employees in Australia or the United States in organizations with at least five employees and who sometimes worked in a team (defined as “a group of at least three individuals who work together on shared tasks or projects”). I only used the 80.7% of respondents who answered “disagree” or “strongly disagree” to an absurd item designed to check for careless responding, devoted on average ≥ 1 s per question, and did not give identical answers to every CCS/CTS item. The resulting sample included 1,640 respondents (60.1% female; 77.5% White, 6.2% Asian, 5.4% Black, 12.0% other or unreported; *M* age = 40.6, *SD* = 12.2). Respondents also indicated how long they had been with the team or organization being described: The percentage of respondents who had been with their organization or team for 1 year or less was, respectively, 13.6 and 25.4%; between 1 and 6 years, 45.3 and 49.4%; and 6 or more years, 41.1 and 25.2%. Thus, unsurprisingly, respondents typically had been with organizations longer than teams.

#### Materials and Procedure

Participants completed an online questionnaire. First, participants completed (randomly) either the CCS (*n* = 808) or CTS (*n* = 832). The CTS differed from the CCS in only two ways: The CTS instructions asked participants to describe the “team you work with most often” (instead of “organization where you work”) and the CTS item stems were “In this team…” (instead of “In this organization…”). Next, to begin investigating validity, participants completed additional measures of group culture and performance; when completing these additional measures, participants who completed the CCS were asked to rate their organization and participants who completed the CTS were asked to rate their team.

Specifically, some (*n* = 544) participants completed the *Organizational Culture Assessment Instrument* (OCAI; Cameron and Quinn, 2011), which measures Clan, Adhocracy, Market, and Hierarchy cultures. Clan cultures are like a family in which members feel trust and loyalty to each other; the culture emphasizes collaboration, consensus, and nurturing members’ potentials. Adhocracy cultures are dynamic, innovative, and experimental; the culture emphasizes taking risks, exploring opportunities, developing new products or services, and being cutting-edge. Market cultures are demanding, no-nonsense, and results-oriented; the culture emphasizes competing aggressively, achieving tough measurable goals, and winning in the marketplace. Hierarchy cultures are organized around conformity to formal rules, stable structures, and clear policies; the culture emphasizes smooth efficiency, predictable processes and outcomes, and stable jobs and relationships.

The other (*n* = 1,096) participants completed 20 items assessing Agreeableness (e.g., “cooperative”) and Extraversion (e.g., “talkative”) from Hofmann and Jones (2005) *Measure of Collective Personality* (MCP), whose items were derived from Goldberg’s (1992) five-factor indicators of individual personality.^3^ Following Hofmann and Jones, participants indicated whether items described “the character of this organization [or team] … that is, how its members behave” on five-point scales ranging from *Strongly Disagree* to *Strongly Agree*. (If participants gave identical answers to every MCP item, then I omitted their MCP responses from the analyses, but—since the MCP was at the end of the questionnaire when participants may have felt impatient or fatigued—I still used their CCS/CTS responses).

Finally, all participants rated the degree to which “I am satisfied with my experience as a member of this organization [team]” and “This organization [team] satisfies the needs of its customers/clients” on five-point (*Strongly Disagree* to *Strongly Agree*) scales. The two moderately correlated items (*r* = 0.62) were averaged to yield a measure of overall satisfaction with the group. Descriptive statistics and interscale correlations for the OCAI, MCP, and Satisfaction scales are reported in Supplementary Tables S1, S2 of the Supplementary Materials.

### Results and Discussion

#### Psychometric and Circumplex Properties

Table 3 reports the CCS/CTS octant scales’ descriptive statistics, internal reliabilities, and intercorrelations; the results mirror those in Study 1. Internal consistencies were good (ranging from 0.72 to 0.92), and greater for scales anchoring the communal dimension (LM and DE) than scales anchoring the agentic dimension (PA and HI). Standard deviations also tended to be greater for the communal/uncommunal than the agentic/unagentic scales. People tended to describe their workplace norms as much more communal (e.g., LM) than uncommunal (e.g., DE) and somewhat more agentic (e.g., PA) than unagentic (e.g., HI).

**Table 3:** CCS and CTS octant scale descriptive statistics, reliabilities, intercorrelations, and loadings on communal and agentic principal components—Study 2.

	CCS			CTS			Correlations								Communal loading		Agentic loading	
Octant	α	*M*	*SD*	α	*M*	*SD*	PA	BC	DE	FG	HI	JK	LM	NO	CCS	CTS	CCS	CTS
PA	0.77	2.42	0.64	0.74	2.49	0.61		0.48	0.03	−0.16	−0.20	−0.05	0.16	0.53	0.18	0.00	0.79	0.74
BC	0.88	1.90	0.86	0.84	1.75	0.82	0.44		0.64	0.39	0.04	−0.42	−0.44	−0.08	−0.65	−0.70	0.52	0.44
DE	0.92	1.25	0.96	0.92	1.00	0.91	−0.15	0.59		0.73	0.30	−0.38	−0.69	−0.45	−0.91	−0.89	−0.08	−0.09
FG	0.84	1.44	0.76	0.86	1.30	0.76	−0.40	0.25	0.69		0.59	−0.07	−0.46	−0.53	−0.67	−0.70	−0.55	−0.49
HI	0.76	1.87	0.66	0.72	1.77	0.64	−0.35	−0.17	0.14	0.52		0.43	−0.02	−0.24	−0.01	−0.17	−0.78	−0.73
JK	0.85	2.19	0.78	0.80	2.37	0.71	−0.12	−0.56	−0.52	−0.11	0.49		0.61	0.31	0.71	0.62	−0.51	−0.48
LM	0.90	2.65	0.84	0.88	2.93	0.73	0.29	−0.45	−0.81	−0.56	0.00	0.62		0.67	0.92	0.87	0.09	0.03
NO	0.84	2.62	0.70	0.81	2.83	0.61	0.60	−0.11	−0.58	−0.62	−0.27	0.29	0.72		0.71	0.63	0.52	0.51

*Circumplex Culture Scan (CCS), n = 808; Circumplex Team Scan (CTS), n = 832. PA, courageous and pushy; BC, competitive and combative; DE, rude and guarded; FG, evasive and hesitant; HI, timid and cautious; JK, yielding and Modest; LM, respectful and open; NO, engaged and confident. Ratings were on Strongly Disagree (0) to Strongly Agree (4) scales. α, Cronbach’s alpha. In the correlation matrix, CCS scale intercorrelations appear below the diagonal and CTS scale intercorrelations appear above the diagonal. Correlations > 0.09 are significant at p < 0.01. The factor loadings reflect Procrustean rotation aligning the first two principal components with the theoretical orientations of the communal and agentic dimensions.*

Exploratory factor analyses showed that the first two factors explained 70.0 and 62.8% of CCS and CTS scale variance, respectively. The third factor was a response elevation factor on which all octants loaded positively (eigenvalues = 0.85 and 1.31 for the CCS and CTS, respectively). The remaining factors explained trivial amounts of variance (eigenvalues < 0.15).

**FIGURE 2:**
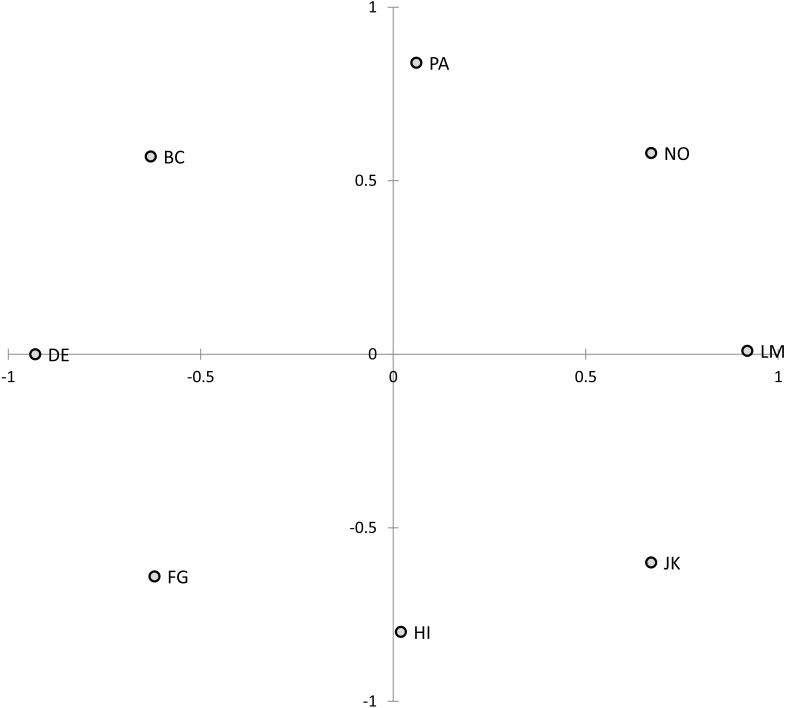
Structure of the Circumplex Culture Scan (CCS) and Circumplex Team Scan (CTS) scales (Study 2). Solution rotated for maximum convergence to theoretical angular locations. PA, courageous and pushy; BC, competitive and combative; DE, rude and guarded; FG, evasive and hesitant; HI, timid and cautious; JK, yielding and modest; LM, respectful and open; NO, engaged and confident.

Principal components analyses showed the first two (i.e., the circumplex) components explained 74.2% of CCS scale variance (communal axis: 44.8%; agentic axis: 29.4%) and 67.0% of CTS scale variance (communal axis: 41.7%; agentic axis: 25.2%). As Table 3 shows, the scales’ loadings revealed the expected sinusoidal pattern: on the communal component, LM and (to a lesser degree) adjacent octants had positive loadings, whereas DE and (to a lesser degree) adjacent octants had negative loadings; on the agentic component, PA and (to a lesser degree) adjacent octants had positive loadings, whereas HI and (to a lesser degree) adjacent octants had negative loadings. The octant scales accordingly formed a circular pattern when plotted within that two-dimensional space (see Figure 2).

The circumplex structure of the CCS/CTS was evaluated using the same procedure as in Study 1. The CCS/CTS showed good fit to the circumplex model’s inequality constraints: The number of order predictions met (out of 288) was 281 for the CCS and 274 for the CTS, *CI*s = 0.95 and 0.90, *p*-values < 0.0005. The CCS/CTS also showed marginally acceptable fit to the more stringent equality constraints: For the equal spacing model, *RMSEA*s for the CCS and CTS = 0.14 and 0.13, 90% *confidence intervals* = [0.13, 0.16] and [0.12, 0.15], *CFI*s = 0.94; for the equal radius model, *RMSEA*s = 0.15 [0.14, 0.16] and 0.13 [0.12, 0.15], *CFI*s = 0.94; and for the equal radius and spacing model, *RMSEA*s = 0.13 [0.12, 0.14] and 0.11 [0.10, 0.13], *CFI*s = 0.93 and 0.94.

#### Convergent and Discriminant Validity

Because the CCS and CTS produced similar results, for simplicity, the following analyses combined the data from the CCS and CTS (Supplementary Table S3 in the Supplementary Materials reports the results for the CCS and CTS separately). Table 4 (left side) shows the zero-order correlations of the OCAI, MCP, and Satisfaction scales with each CCS/CTS octant. The OCAI-Clan and MCP-Agreeable scales (reflecting cooperative, collaborative cultures) showed strong positive correlations with CCS/CTS communal (LM and adjacent) octants, strong negative correlations with CCS/CTS uncommunal (DE and adjacent) octants, and weak associations (−0.2 < *r* < 0.2) with the agentic and unagentic (PA and HI) octants. The OCAI-Market scale (reflecting distinctively demanding, competitive cultures) showed the opposite pattern. The OCAI-Adhocracy scale (reflecting forward-looking risk-taking cultures) showed positive correlations with CCS/CTS agentic (PA and adjacent) octants. The MCP-Extraverted scale (reflecting confident, energetic, interactive cultures) showed positive correlations with CCS/CTS agentic-and-communal (NO and adjacent) octants, negative correlations with CCS/CTS unagentic-and-uncommunal (FG and adjacent) octants, and weak associations with the remaining octants. The OCAI-Hierarchy scale (reflecting structured, rule-governed cultures) showed the opposite pattern. In sum, the patterns of correlations between the CCS/CTS and OCAI or MCP scales supported the CCS/CTS octant scales’ convergent and discriminant validity.

**Table 4:** Correlations and summary parameters for relations between the CTS/CCS and Organizational Culture Assessment Inventory (OCAI), Measure of Collective Personality (MCP), and satisfaction scales—Study 2.

	Correlations with CCS/CTS octant scales								Summary parameters					
Variables	PA	BC	DE	FG	HI	JK	LM	NO	Elevation [CI]	Communal vector [CI]	Agentic vector [CI]	Angle [CI]	Amplitude [CI]	*R*^2^
**OCAI**														
Clan	−0.04	−0.45	−0.47	−0.32	−0.09	0.35	0.50	0.36	−0.02 [−0.04, 0.01]	0.50 [0.44, 0.56]	−0.01 [−0.07, 0.05]	359.1 [351.9, 6.0]	0.50 [0.44, 0.57]	0.98
Adhocracy	0.24	0.16	−0.07	−0.10	−0.12	−0.04	0.08	0.13	0.04 [0.01, 0.06]	0.04 [−0.05, 0.12]	0.17 [0.10, 0.22]	76.0 [52.0, 106.5]	0.17 [0.11, 0.24]	0.92
Market	0.11	0.49	0.47	0.27	0.03	−0.35	−0.48	−0.29	0.03 [0.00, 0.06]	−0.49 [−0.56, −0.41]	0.07 [0.00, 0.13]	171.9 [164.0, 179.8]	0.49 [0.42, 0.56]	0.98
Hierarchy	−0.22	−0.03	0.16	0.18	0.16	−0.06	−0.19	−0.24	−0.03 [−0.05, −0.00]	−0.17 [−0.25, −0.08]	−0.16 [−0.23, −0.09]	224.6 [207.1, 244.9]	0.23 [0.16, 0.31]	0.99
**MCP**														
Agreeableness	0.16	−0.49	−0.81	−0.57	−0.10	0.54	0.80	0.57	0.01 [−0.01, 0.04]	0.79 [0.76, 0.82]	0.08 [0.03, 0.14]	6.10 [2.1, 10.1]	0.79 [0.77, 0.83]	1.00
Extraversion	0.49	−0.06	−0.50	−0.64	−0.44	0.07	0.50	0.63	0.01 [−0.02, 0.03]	0.50 [0.45, 0.55]	0.44 [0.40, 0.49]	41.80 [37.0, 46.3]	0.67 [0.63, 0.70]	1.00
**Satisfaction**	0.19	−0.22	−0.45	−0.39	−0.14	0.25	0.50	0.41	0.02 [0.00, 0.04]	0.46 [0.41, 0.51]	0.14 [0.11, 0.17]	17.1 [12.9, 21.1]	0.48 [0.40, 0.53]	1.00

*Ns = 544 OCAI, 869 MCP, 1596 Satisfaction. PA, courageous and pushy; BC, competitive and combative; DE, rude and guarded; FG, evasive and hesitant; HI, timid and cautious; JK, yielding and modest; LM, respectful and open; NO, engaged and confident. Correlations > 0.09 are significant at p < 0.01. R^2^ = goodness of fit to ideal curve. CI, confidence intervals computed using resampling procedures implemented by the circumplex package for R (Girard et al., 2018).*

#### Circumplex Summary Parameters

**FIGURE 3:**
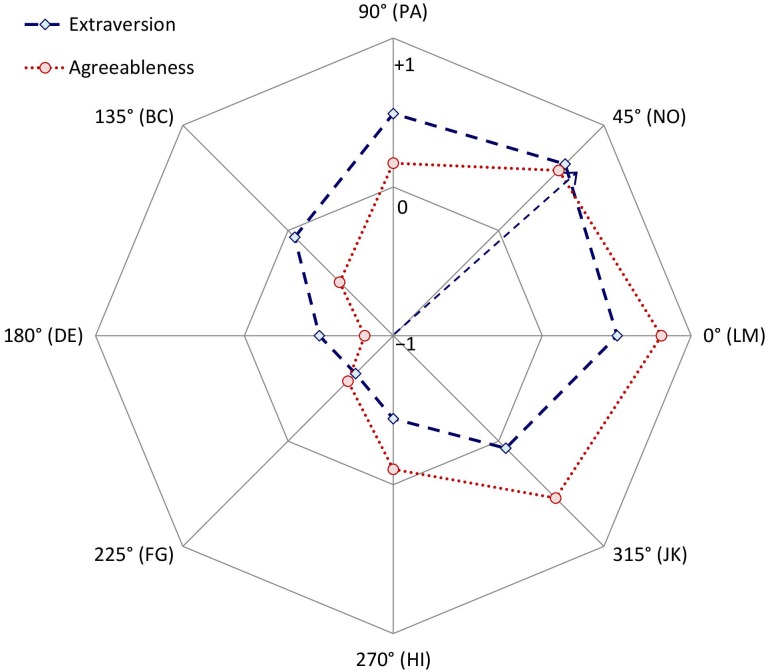
Correlations between collective Agreeableness or Extraversion and each Circumplex Team Scan octant scale. Within each octant, correlations could range from a minimum of –1 (at the circle’s midpoint) to a maximum of +1 (at the circumference). The arrow shows the vector sum of the Extraversion correlations.

A distinguishing asset of circumplex inventories is that understanding how other variables correlate with the octants may not require examining the correlations with each octant individually. Instead, if certain conditions are met, then profiles of correlations with octant scales (like those examined in the preceding paragraph) can be depicted by a smaller set of circumplex “summary parameters.” For example, consider the correlations between the CCS/CTS and MCP Agreeableness and Extraversion scales in Table 4. Figure 3 displays these correlations on the circumplex: Along each octant scale, more negative correlations are closer to the midpoint of the circle and more positive correlations are closer to the circumference. Figure 3 highlights how both Agreeableness and Extraversion correlated positively with communal (and negatively with uncommunal) norms, while Extraversion correlated more positively with agentic (and more negatively with unagentic) norms than did Agreeableness.

**FIGURE 4:**
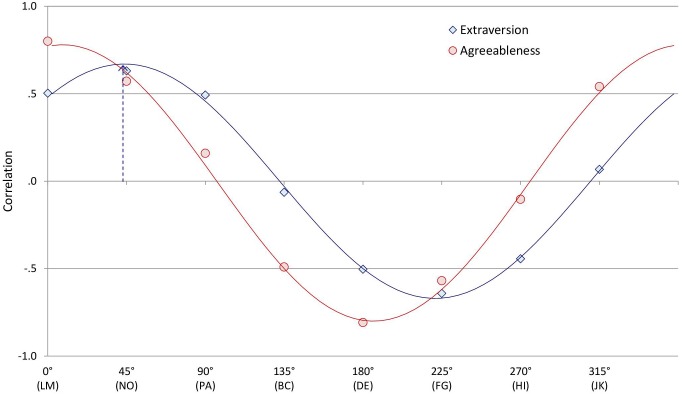
Circles and diamonds indicate the observed correlations between collective Agreeableness or Extraversion and each Circumplex Team Scan octant scale. Continuous lines show the best-fitting cosine curve for each profile of correlations. The arrow shows the vector sum of the profile of correlations with Extraversion.

To show how these profiles of correlations can be summarized using trigonometry, Figure 4 illustrates a different way to represent these same data. Specifically, Figure 4 unrolls Figure 3 so that each octant scale extends upward from successive points along the horizontal axis, which is how a wave function is typically depicted. Because the pattern of correlations among the CCS/CTS scales meets the criteria for a circumplex, to the degree that an external variable (e.g., Extraversion) is associated with agentic and communal social norms, its pattern of correlations with the CCS/CTS octant scales should conform to a sinusoidal wave function or cosine curve that can be summarized with just a few trigonometric parameters (Gurtman and Pincus, 2003). The external variable’s average correlation across all octant scales is the curve’s elevation; CCS/CTS elevations lack substantive meaning and, as expected, showed no noteworthy associations with the other measures. The external variable’s average correlations with the horizontal communal vector (*X*) and vertical agentic vector (*Y*) of the circumplex are

(1) $$\begin{array}{c} X=0.25*[\text{LM}+0.707*(\text{JK}+\text{NO})\\ \;-\text{DE}-0.707*(\text{BC}+\text{FG})] \end{array}$$

(2) $$\begin{array}{c} Y=0.25*[\text{PA}+0.707*(\text{BC}+\text{NO})\\ \;-\text{HI}-0.707*(\text{JK}+\text{FG})] \end{array}$$

where PA is the correlation with the PA octant, BC the correlation with the BC octant, and so on. The (*X*, *Y*) coordinate or vector sum defined by these two vectors shows how far and at what angle the external variable projects onto the circumplex space. For example, the arrow in Figure 3 shows Extraversion’s projection onto the CCS/CTS circumplex as a summary vector; the arrow in Figure 4 shows the same vector, with its *angle* being the location of the wave’s peak on the horizontal axis and its length being the wave’s *amplitude* (i.e., the distance between its elevation and its peak) on the vertical axis. Amplitude reflects the degree to which a construct is linked to relatively high scores (i.e., strong group norms) in one circumplex region and relatively low scores in the opposing circumplex region.

A profile’s vector angle and amplitude are meaningful only to the degree that the profile does in fact fit a cosine curve. For example, in Figure 4, while the points show the observed correlations between Extraversion or Agreeableness and each octant, the lines show the associated best-fitting curves (computed using Equation 1 from Zimmermann and Wright, 2017). The degree to which observed correlations deviate from the ideal cosine curve is summarized in a goodness-of-fit index, *R*^2^ (for details, see Gurtman and Pincus, 2003). By convention, good fit is defined as *R*^2^ values ≥ 0.8 (Boudreaux et al., 2018). As Table 4 (last column) shows, all the scales’ profiles showed excellent fit (*R*^2^ values ≥ 0.95). For example, the Extraversion and Agreeableness scales showed almost perfect (*R*^2^ > 0.99) conformity to their ideal cosine curves (a conclusion corroborated simply by “eye-balling”; Figure 4). Thus, each profile of correlations listed in Table 4 can be effectively summarized by its communal vector, agentic vector, and overall vector amplitude and angle (see Table 4, right side).

#### Projecting the OCAI and MCP Onto the IPC

**FIGURE 5:**
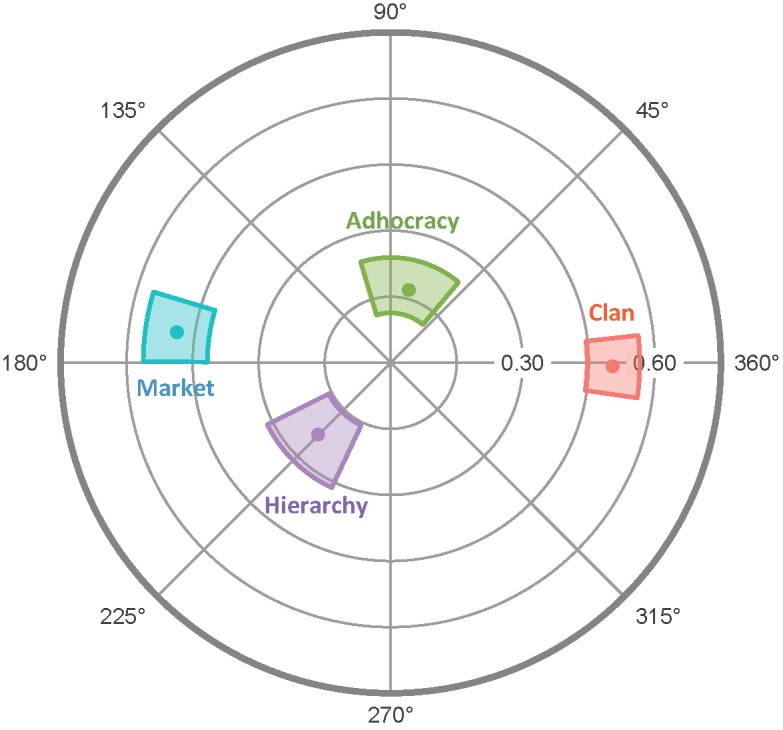
Radar chart of OCAI scales’ projections on the CCS/CTS circumplex. Dots represent mean values and colored regions represent bootstrapped 95% confidence intervals (computed and plotted using the *circumplex* package for R; Girard et al., 2018).

**FIGURE 6:**
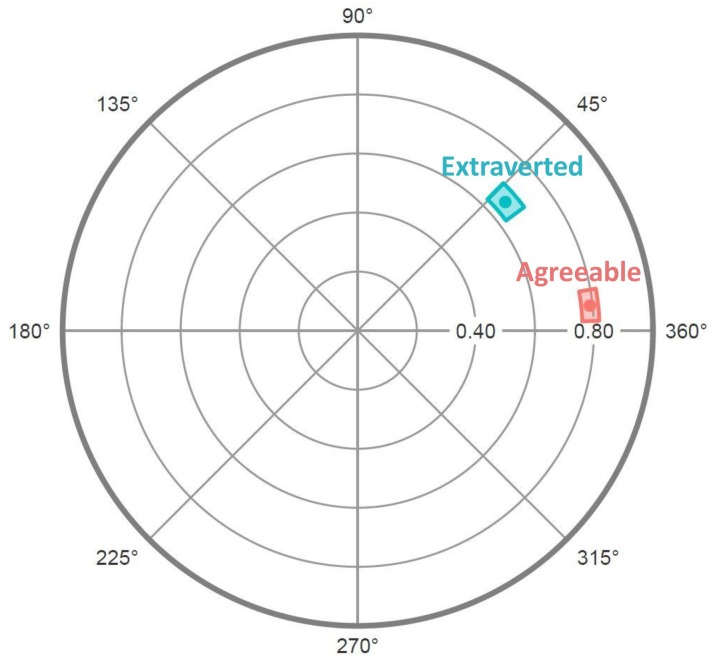
Radar chart of MCP scales’ projections on the CCS/CTS circumplex. Dots represent mean values and colored regions represent bootstrapped 95% confidence intervals (computed and plotted using the *circumplex* package for R; Girard et al., 2018).

To summarize and visualize the results, I plotted the endpoints of the vectors for the OCAI scales in Figure 5 and the MCP scales in Figure 6. Recall that the OCAI assesses four culture types: Clan (emphasizing shared loyalty, trust, and cooperation), Adhocracy (emphasizing risk-taking and dynamism), Market (emphasizing surpassing demanding objective standards), and Hierarchy (emphasizing clear stable policies and social structures). Each culture reflects a different quadrant of the two-dimensional Competing Values Framework model (Cameron and Quinn, 2011). Past research generally finds that cultures reflecting opposing quadrants (i.e., Clan versus Market and Hierarchy versus Adhocracy) have opposing relationships with other variables. As Figure 5 shows, that was also true in the current data. Specifically, whereas Clan cultures were moderately positively associated with communal norms, the opposite was true of Market cultures, and (to a lesser degree) whereas Adhocracy was positively associated agentic norms, Hierarchy was negatively associated with agentic and communal norms. In sum, in terms of the Competing Values Framework’s (integrated–differentiated and stable–flexible) dimensions, it tended to be that communal norms were most prevalent in flexible integrated cultures, uncommunal norms were most prevalent in stable differentiated cultures, agentic norms were most prevalent in flexible differentiated cultures, and unagentic norms were most prevalent in stable integrated cultures.

As Figure 6 shows, Agreeableness (e.g., *helpful* versus *selfish*) and Extraversion (e.g., *talkative* versus *timid*) showed very pronounced and distinct projections onto the CCS/CTS circumplex, presumably because these were the scales that most directly assessed interpersonal behavior. Extraversion and Agreeableness are considered the “interpersonal” factors of the five-factor model of personality, and studies of individual personality (e.g., McCrae and Costa, 1989; DeYoung et al., 2013) have repeatedly found them to load strongly on the IPC in ways that roughly mirror the current findings for collective personality. Specifically, in the current data, communal group norms were moderately positively associated with Extraversion and strongly positively associated with Agreeableness. In contrast, agentic norms were moderately positively associated with Extraversion, but at most weakly positively associated with Agreeableness. Thus, on average, Agreeable cultures were located in the Open [M] segment and Extraverted cultures were located in the Engaged-Confident [NO] octant. Finally, like Agreeableness, Satisfaction was positively associated with communal and—to a lesser degree—agentic norms, also placing its overall projection in the “Open” [M] segment of the circle.

#### Incremental Validity

Regression of Satisfaction on the CCS/CTS vectors confirmed that communal and agentic norms explained significant variance in Satisfaction: for participants who completed the MCP, *b* Communal = 0.63 (*SE* = 0.04), *b* Agentic = 0.19 (*SE* = 0.06), *R*^2^ = 0.25; for participants who completed the OCAI, *b* Communal = 0.66 (*SE* = 0.04), *b* Agentic = 0.21 (*SE* = 0.06), *R*^2^ = 0.41; all *p* values < 0.001. However, the MCP and OCAI also predicted Satisfaction (*R*^2^ values = 0.19 and 0.30, respectively). To test if the CCS/CTS explained unique variance in Satisfaction after controlling for the variance explained by the MCP or OCAI, I repeated the preceding regressions after first adding the MCP or OCAI scales. After controlling for the variance shared with the MCP: *b* Communal = 0.16 (*SE* = 0.07), *b* Agentic = 0.23 (*SE* = 0.07), Δ*R*^2^ = 0.01, *F*Δ(2,852) = 7.29, *p* ≤ 0.001. After controlling for the variance shared with the OCAI: *b* Communal = 0.58 (*SE* = 0.04), *b* Agentic = 0.25 (*SE* = 0.06), Δ*R*^2^ = 0.23, *F*Δ(2,535) = 106.62, *p* ≤ 0.001. Thus, the CCS/CTS demonstrated incremental validity.^4^

## Study 3—Field Testing

Organizational consultants administered the CCS to 516 respondents from 21 different organizations (*M n* per organization = 24.6, *SD* = 22.9) and the CTS to 347 respondents from 38 teams (*M n* per team = 9.1, *SD* = 5.1) based in Australia, yielding a total of 863 respondents (*M* age = 40.9, *SD* = 10.6; 61.1% male, 37.8% female, 2.8% unreported; 78.2% Caucasian, 10.2% Asian, 4.2% unreported, 7.4% other; 63% had earned a bachelor level or higher degree, and 44% were in some type of managerial role). The percentage of respondents who had been with their organization or team for ≤1 year was, respectively, 13.8 and 28.7%; between 1 and 6 years, 44.3 and 49.7%; and ≥6 years, 41.9 and 21.6%. Thus, as in Study 2, respondents had more history with an organization than a team.

**Table 5:** Within-group interrater agreement (*r*_wg_, *a*_wg_) and intraclass correlation (ICC) aggregation indices.

Target/scale	*r_WG(J)_·_uniform_*	*r_WG(J)_·_normal_*	*a_WG(J)_*	ICC(2)	ICC(1)	*F* ratio
**Organizations**						
PA	0.94	0.81	0.68	0.66	0.07	2.92^∗∗^
BC	0.92	0.68	0.62	0.73	0.10	3.67^∗∗^
DE	0.90	0.49	0.50	0.76	0.12	4.22^∗∗^
FG	0.93	0.74	0.64	0.74	0.10	3.84^∗∗^
HI	0.93	0.69	0.64	0.77	0.12	4.35^∗∗^
JK	0.92	0.67	0.62	0.56	0.05	2.25^∗∗^
LM	0.92	0.66	0.60	0.83	0.17	5.94^∗∗^
NO	0.94	0.75	0.65	0.76	0.12	4.25^∗∗^
Satisfaction	0.80	0.52	0.60	0.69	0.08	3.26^∗∗^
**Teams**						
PA	0.94	0.73	0.68	0.65	0.17	2.82^∗∗^
BC	0.92	0.63	0.62	0.77	0.27	4.43^∗∗^
DE	0.92	0.73	0.59	0.87	0.41	7.45^∗∗^
FG	0.94	0.75	0.67	0.83	0.35	5.87^∗∗^
HI	0.92	0.62	0.63	0.74	0.24	3.92^∗∗^
JK	0.92	0.63	0.62	0.55	0.12	2.22^∗∗^
LM	0.93	0.68	0.61	0.83	0.35	5.92^∗∗^
NO	0.94	0.76	0.69	0.80	0.31	5.07^∗∗^
Satisfaction	0.81	0.58	0.59	0.84	0.37	6.35^∗∗^

*N = 347 CTS respondents from 38 teams, and 516 CCS respondents from 21 organizations. PA, courageous and pushy; BC, competitive and combative; DE, rude and guarded; FG, evasive and hesitant; HI, timid and cautious; JK, yielding and modest; LM, respectful and open; NO, engaged and confident. ^∗∗^p < 0.005.*

### Materials and Procedure

Respondents completed an online survey at their workplace. First, to assess their team or organization’s actual norms, respondents completed the CTS or CCS (using the same version as in Study 2). Second, respondents completed the two satisfaction items used in Study 2 (interitem *r* = 0.49), which were averaged to yield an index of overall satisfaction with the group.^5^ Third, most respondents (*n* = 729) completed the CCS or CTS again, but this time indicated what they believed would be the ideal norms for their organization or team—i.e., how members “ideally would” behave. Ideal social norms were measured primarily for use in feedback sessions with the participating groups, where identifying gaps between ideal and actual norms could help stimulate and focus efforts to improve organizational/team culture.

### Data Aggregation

The CCS/CTS scales were designed to measure characteristics of a group (i.e., its norms) by assessing and then aggregating group members’ judgments of those group characteristics. In other words, the CCS/CTS scales measure “referent-shift composition constructs” (Chan, 1998). To evaluate whether members’ aggregated judgments do reflect meaningful group-level constructs (Klein and Kozlowski, 2000), Table 5 reports standard indices of the degree to which the CCS/CTS scales show consensus within groups and differentiation across groups.

Specifically, within-group interrater agreement was assessed using the *r*_wg(j)_ index, which compares observed variance among group members to the variance expected if respondents were responding randomly (James et al., 1993). We estimated *r*_wg(j)_ assuming two different random response distributions: a uniform distribution, which is how *r*_wg(j)_ is most often computed and yields an upper estimate, and a normal distribution, which is more realistic and yields a more conservative estimate (LeBreton and Senter, 2008). Assuming a uniform random distribution, within both organizations and teams, the average *r*_wg(j)_ was 0.93 and exceeded 0.90 for all scales, indicating very strong agreement. Assuming a normal random distribution, within both organizations and teams, the average *r*_wg(j)_ was 0.69, and all scales (except for “DE” when rating organizations) showed moderate to strong agreement (LeBreton and Senter, 2008). In sum, the observed agreement levels generally exceeded both the standard criterion for aggregation and the values typically reported in the literature (Woehr et al., 2015).

We also quantified within-group agreement using *a*_wg(j)_ (Brown and Hauenstein, 2005), which is a function of the ratio of the observed within-group variance to the maximum possible variance. Although *r*_wg_ is more popular, *a*_wg_ has the advantages of not being influenced by sample size or requiring assumptions about the response distribution. Values of *a*_wg(j)_ can range from −1.0 to 1.0, with values >0.6 indicating acceptable agreement (Brown and Hauenstein). As Table 5 shows, the average *a*_wg(j)_ was 0.62 within organizations and 0.63 within teams, and exceeded 0.60 for all scales except “DE” and Satisfaction.

Intraclass correlations (ICCs) were calculated from one-way random-effects ANOVAs, with group membership as the between-participants variable. The ICC(2) values shown in Table 5 indicate that group means reliably differentiated between groups. The ICC(2) values for all scales except JK and PA exceeded 0.70 (the standard criterion for “good reliability”), and the average ICC(2) was 0.73 for organizations and 0.76 for teams, which exceeds the average ICC(2) values reported for teams (0.64) and organizations (0.70) in the literature (Woehr et al., 2015). The ICC(1)s and associated *F*-values indicate that group membership explained a significant proportion of the variance in every CCS and CTS scale (all *p*-values < 0.005). Teams explained a larger percentage of variance (*M* = 28%, range = 12–41%) than did organizations (*M* = 11%, range = 05–17%).

In sum, there was sufficiently reliable within-group consistency and between-group variance to justify aggregating octant scores across individuals within groups. Thus, in the results below, the unit of analysis is the group (e.g., CCS/CTS octant scores were averaged across team members or organization members prior to analysis).

### Results and Discussion

#### Circumplex Structure

**FIGURE 7:**
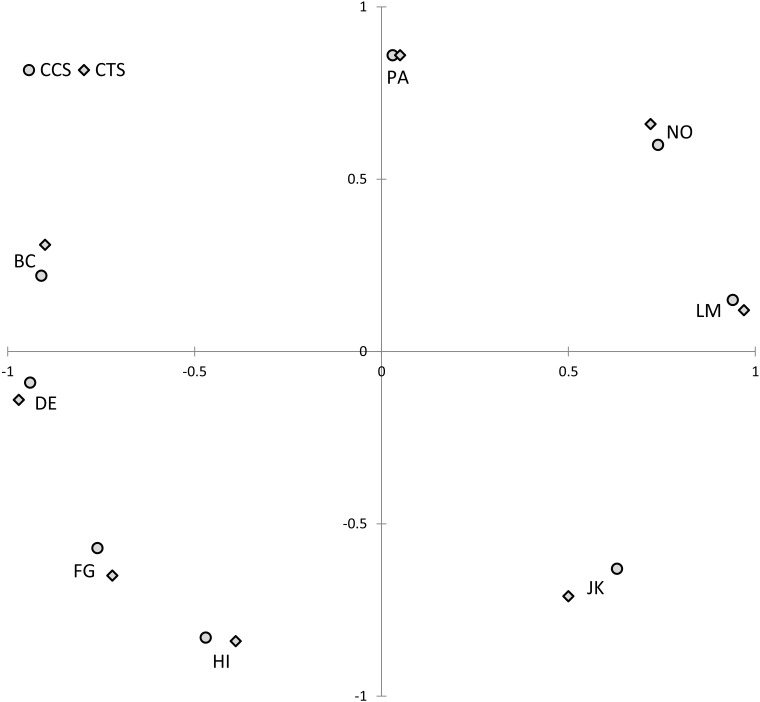
Structure of the Circumplex Culture Scan (CCS) and Circumplex Team Scan (CTS) scales (Study 3). Solution rotated for maximum convergence to theoretical angular locations. PA, courageous and pushy; BC, competitive and combative; DE, rude and guarded; FG, evasive and hesitant; HI, timid and cautious; JK, yielding and modest; LM, respectful and open; NO, engaged and confident.

Factor analyses showed that most CCS and CTS scale variance was explained by the first two factors (eigenvalues ranged from 2.13 to 4.72), with the remaining factors explaining little (eigenvalues < 0.5). The CCS/CTS scales’ principal components analysis loadings are reported in Supplementary Table S4 (along with descriptive statistics) and plotted in Figure 7. The circumplex structure of the CCS/CTS was formally evaluated using the same procedures as in the previous studies. Conformity to the circumplex model’s inequality constraints was acceptable: The number of predictions met (out of 288) was 259 for the CCS and 264 for the CTS, *CI*s = 0.80 and 0.83, *p*-values < 0.0005. However, conformity to the equality constraints—especially equal spacing—did not meet standard criteria: For the equal spacing model, *RMSEA*s for the CCS and CTS = 0.28 [0.18, 0.38] and 0.27 [0.20, 0.34], *CFI*s = 0.84 and 0.87; for the equal radius model, *RMSEA*s = 0.21 [0.09, 0.31] and 0.24 [0.17, 0.31], *CFI*s = 0.90 and 0.91; and for the equal radius and equal spacing model, *RMSEA*s = 0.26 [0.17, 0.35] and 0.27 [0.21, 0.33], *CFI*s = 0.83 and 0.84. Collectively, these tests indicate that the scales, while conforming to a circular model, deviated from an ideal equally spaced circumplex.

Visually, in Figure 7, conformity to the inequality constraints is evident in the octants being in the correct order, while non-conformity to the equality constraints is evident in the octants not showing equal spacing and communalities. Technically, the current data therefore met “quasi-circumplex” but not “circulant” criteria (Guttman, 1954), meaning that the standard weights for computing communal and agentic vectors (in Equations 1 and 2) are not the optimal weights for this data set. Pragmatically, though, if IPC scales at least exceed quasi-circumplex criteria, then deviations from perfect circulant criteria should have minimal impact (Gurtman and Pincus, 2003). Therefore, to prevent overfitting and promote cumulative replicable findings, the analyses below employed the standard formulas for computing communal and agentic vectors.

**Table 6:** Correlations and summary parameters for relations between CTS/CCS and satisfaction—Study 3.

	Correlations with satisfaction								Summary parameters					
Variables	PA	BC	DE	FG	HI	JK	LM	NO	Elevation [CI]	Communal vector [CI]	Agentic vector [CI]	Angle [CI]	Amplitude [CI]	*R*^2^
Actual norms														
CCS	0.17	−0.71	−0.74	−0.76	−0.54	0.33	0.73	0.64	−0.11 [−0.20, −0.01]	0.80 [0.51, 0.98]	0.24 [0.07, 0.43]	17.0 [5.1, 32.6]	0.83 [0.57, 1.03]	0.96
CTS	0.27	−0.74	−0.92	−0.77	−0.48	0.30	0.91	0.79	−0.08 [−0.13, −0.04]	0.92 [0.78, 1.01]	0.28 [0.04, 0.54]	16.9 [2.4, 33.0]	0.96 [0.86, 1.05]	0.97
Ideal norms														
CCS	−0.22	−0.25	−0.12	−0.01	0.00	0.07	0.09	0.01	−0.05 [−0.17, 0.07]	0.11 [−0.38, 0.53]	−0.11 [−0.49, 0.28]	315.7 [217.6, 41.2]	0.16 [0.08, 0.66]	0.84
CTS	−0.20	−0.14	−0.16	−0.26	−0.10	−0.09	0.16	0.14	−0.08 [−0.15, 0.00]	0.16 [−0.11, 0.50]	0.04 [−0.27, 0.42]	13.2 [232.1, 77.6]	0.16 [0.05, 0.63]	0.66

*Ns = 21 organizations and 38 teams for the CCS and CTS, respectively. PA, courageous and pushy; BC, competitive and combative; DE, rude and guarded; FG, evasive and hesitant; HI, timid and cautious; JK, yielding and modest; LM, respectful and open; NO, engaged and confident. CCS correlations > 0.44 and CTS correlations > 0.32 are significant at p < 0.05. CI, confidence intervals computed using resampling procedures implemented by the circumplex package for R (Girard et al., 2018).*

#### Satisfaction

Because satisfaction ratings demonstrated sufficient within-group agreement and between-group differentiation to justify treating satisfaction as a group-level variable (see Table 5), the following analyses were conducted on group-level satisfaction (averaged across all members of each team or organization). Satisfaction with organizations (*M* = 2.99, *SD* = 0.29) and teams (*M* = 3.01, *SD* = 0.59) did not differ. The satisfaction scales’ correlations with the CCS/CTS scales showed very good fit to a cosine curve and clear specificity regarding which social norms they were positively or negatively related to (see Table 6, top rows). Therefore, the communal and agentic summary vectors and angles accurately encapsulate the associations between group culture and satisfaction. Specifically, replicating the associations between culture and satisfaction found in Study 2, satisfaction was positively associated with communal and (to a lesser degree) agentic norms, and negatively associated with uncommunal and (to a lesser degree) unagentic norms, placing satisfaction’s overall angle of projection inside the “Open” [M] 16th of the circle.

**Table 7:** Paired samples *t*-tests of actual and ideal social norms—Study 3.

	Actual		Ideal		
Octant	*M*	*SD*	*M*	*SD*	*t*(57)
PA	2.25	0.26	2.62	0.21	−10.81^∗∗^
BC	1.64	0.43	1.38	0.21	4.48^∗∗^
DE	1.08	0.58	0.42	0.29	9.53^∗∗^
FG	1.39	0.36	0.80	0.24	14.82^∗∗^
HI	1.71	0.31	1.34	0.24	10.49^∗∗^
JK	2.22	0.27	2.15	0.27	1.65
LM	2.64	0.51	3.37	0.27	−11.57^∗∗^
NO	2.55	0.37	3.21	0.27	−14.19^∗∗^

*N = 58 teams and organizations (one team did not rate ideal norms). ^∗∗^p < 0.001.*

#### Ideal Norms

As noted earlier, ideal social norms were measured primarily for use in feedback sessions; however, as Table 7 shows, more general comparisons of actual and ideal social norms revealed some interesting patterns. (Because teams and organizations yielded similar results, the reported analyses were conducted across all teams and organizations; separate results for teams and organizations are reported in Supplementary Table S5). First, as in Studies 1 and 2, actual workplace norms were more communal than uncommunal and (to a lesser degree) more agentic than unagentic. Second, groups’ ideal social norms were even more communal and agentic (and less uncommunal and unagentic) than groups’ actual social norms. Third, the variances in actual communal or uncommunal norms exceeded the variances in both ideal norms and actual agentic or unagentic norms (all *p*-values < 0.001), meaning that groups diverge from each other most in their actual communal/uncommunal behaviors and diverge least in their ideals.

**FIGURE 8:**
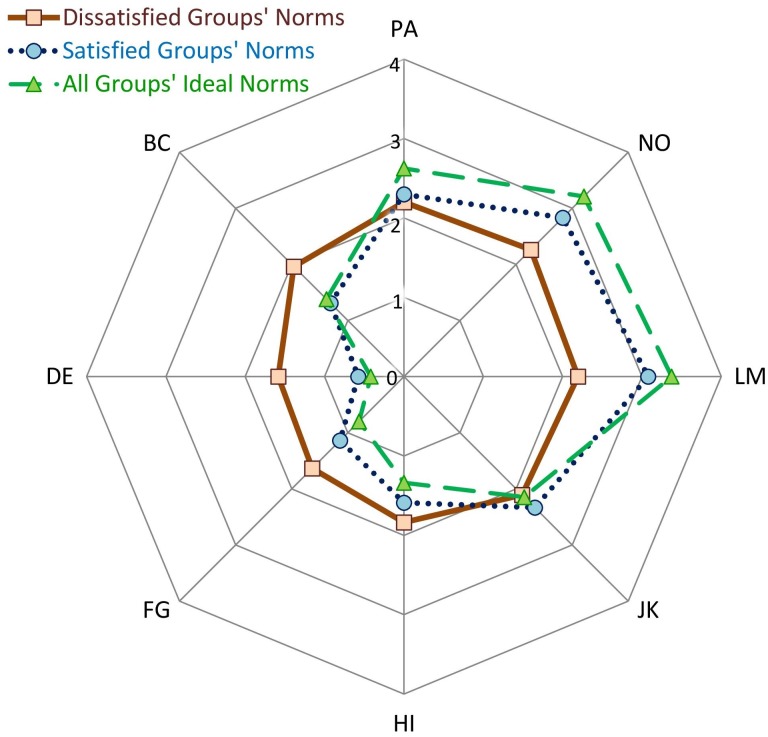
Groups’ ideal norms and the actual norms of relatively satisfied or dissatisfied groups (Study 3). Ratings were made on 0-to-4 scales; thus, along each octant scale, ratings closer to the circumference of the circle indicate stronger endorsement of that type of behavior. PA, courageous and pushy; BC, competitive and combative; DE, rude and guarded; FG, evasive and hesitant; HI, timid and cautious; JK, yielding and modest; LM, respectful and open; NO, engaged and confident.

Associations between ideal norms and satisfaction were not expected; in fact, satisfaction did not have significant associations with the octant scales or with the communal and agentic vectors and accordingly was not associated with any specific region of the ideal norms circumplex (see Table 6, bottom rows). Figure 8 plots the average ideal norms (from Table 7) on the IPC, showing how groups’ ideal norms—regardless of whether the groups were satisfied or dissatisfied—tilted strongly toward being communal and to a lesser degree toward being agentic. Figure 8 also shows the estimated actual norms of groups that were one SD above or below average in satisfaction (computed from the unstandardized coefficients relating satisfaction with each CCS/CTS scale). Whereas the actual norms of satisfied groups were—like ideal norms—tilted strongly toward being communal and to a lesser degree toward being agentic, the actual norms of dissatisfied groups were not. Thus, the discrepancy between actual norms and ideal norms was smaller for satisfied than for dissatisfied groups.

## General Discussion

Social norms, which shape how people work together, are an essential core of organizational and team culture. To develop a broad balanced measure of those group norms, the current project relied on the IPC, which has previously been used to model individuals’ social dispositions. The properties of the resulting Circumplex Culture/Team Scan were then evaluated in online and onsite samples. Below, I summarize the key findings and consider some limitations and opportunities for further investigation.

### Psychometrics

Across all samples, the CCS/CTS octant scales showed acceptable internal consistencies. The scales also showed excellent fit to the circumplex model’s “quasi-circumplex” criteria that scales theoretically closer to each other on the circumplex should empirically be more strongly related to each other. Moreover, in online (but not onsite) samples, the CCS/CTS met the model’s stricter “circulant” criteria that scales theoretically equally close/distant to each other on the circumplex should empirically be equally positively/negatively related to each other.

The CCS/CTS octant scales showed convergent and discriminant validity in relation to other measures of workplace culture. For example, CCS/CTS communal norms were positively associated with agreeable, respectful, collaborative, extraverted, stable, and nurturing (e.g., MCP-Agreeable and OCAI-Clan) cultures, and negatively associated with aggressively competitive (OCAI-Market) or, to a lesser degree, formal (OCAI-Hierarchy) cultures. Agentic norms were positively associated with measures of extraverted, innovative, dynamic (e.g., MCP-Extraverted and OCAI-Adhocracy) cultures, and negatively associated with meticulously structured (e.g., OCAI-Hierarchy) cultures.

Projecting existing measures of organizational culture onto the CCS/CTS circumplex suggests that they may not adequately assess some of the social norms assessed by the CCS/CTS. To the degree that the CCS/CTS reflects different constructs from those assessed by other organizational culture measures, the CCS/CTS may demonstrate incremental validity in predicting various organizational outcomes. Indeed, Study 2 showed that the CCS/CTS explained additional variance in members’ overall satisfaction with their team/organization above and beyond that explained by other culture inventories; therefore, the CCS/CTS does appear to measure norms that other instruments do not. Moreover, the CCS/CTS targets social norms that shape everyday interactions among group members and that consequently may have an especially immediate and direct impact on their everyday functioning and satisfaction.

It bears repeating that the CCS/CTS was designed to complement existing measures of organizational culture, and whether the CCS/CTS or another instrument is appropriate depends on the purpose of the assessment. If the purpose of an assessment includes elucidating the norms shaping the interaction and communication dynamics within a team/organization, then the CCS/CTS may be an appropriate choice. If an assessment focuses on norms that either do not concern social behavior (e.g., valuing detail-focused versus big-picture thinking) or concern behavior toward external agents (e.g., other organizations), then another measure of organizational culture may be more appropriate.

### Descriptive, Ideal, and Injunctive Norms

Within both organizations and teams, people typically reported that agentic norms (courageous and pushy) were somewhat stronger than unagentic norms (timid and cautious), and communal norms (open and respectful) were much stronger than uncommunal norms (rude and guarded). However, there were also differences in the interpersonal cultures of different organizations and teams, especially in the strength of their communal versus uncommunal norms. The more organizations and teams were characterized as having communal and communal-and-agentic norms (e.g., be interactive and expressive) rather than uncommunal norms (e.g., be distancing and competitive), the more they were described as satisfying the needs of their members and their customers/clients.

When assessing current culture or *descriptive norms* in Studies 1–3, the referent of CCS/CTS items was the *behavior* of members within the team or organization. If the CCS/CTS instead assessed *injunctive norms*, then the referent would be the *perceived attitudes* of members of the team or organization toward behaviors from each IPC region. Lacking direct access to others’ attitudes, respondents are prone to infer injunctive norms from descriptive norms (e.g., “if most team members occasionally ridicule others’ contributions, then most team members must approve of that behavior”) (Anderson and Dunning, 2014; Miller and Prentice, 2016). Therefore, when testing and providing feedback to teams and organizations in Study 3, we assessed *ideal* (rather than injunctive) norms, with the referent being respondents’ *own attitudes* (rather than others’ attitudes) toward behaviors from each IPC region.

The typical respondent’s vision of an ideal organizational or team culture was strongly tilted toward being more communal than uncommunal and slightly tilted toward being more agentic than unagentic, and thus closely resembled the actual culture of highly satisfied organizations and teams. This suggests that identifying and narrowing gaps between organizations’ or teams’ current culture and ideal culture could help them improve their outcomes. If injunctive norms (i.e., beliefs about others’ ideal norms) were accurate, then they would perfectly mirror a typical member’s ideal norms. Unfortunately, injunctive norms are often inaccurate. Because people infer injunctive norms from descriptive norms, members of dissatisfied groups may be vulnerable to “pluralistic ignorance” (Halbesleben et al., 2007) and mistakenly conclude that most members favor the current norms. Consequently, it may promote culture change to show group members that most of their peers endorse ideals that are closer to their own ideals and farther from the group’s current norms than they realized.

### Assessing Groups Versus Individuals

The CCS/CTS is a distinctive addition to the family of IPC inventories. Whereas all previous IPC inventories assess *characteristics of individuals* (such as an individual’s traits, goals, or sensitivities), the CCS/CTS assesses *characteristics of groups*.^6^ Any judgments of a group by its members will naturally vary due to members’ distinctive dispositions and positions within the group. Nonetheless, members’ CCS/CTS ratings demonstrated robust within-group agreement and reliable between-group differences. The narrow practical implication is that individuals’ CCS/CTS responses can be treated as indicators of a group-level construct—namely, their organization’s or team’s interpersonal culture. The broader theoretical implication is that a circumplex defined by the dimensions of agency and communion can effectively model not only the personalities of individuals but also the personalities of entire teams or organizations.

That said, different dynamics shape individuals versus groups. One such dynamic is *interpersonal complementarity*—the tendency for interpersonal actions to invite reactions that are similar in communion but dissimilar in agency (Horowitz et al., 2006; Sadler et al., 2011). That is, communal actions (e.g., welcoming and supporting) invite similarly communal reactions from others and uncommunal actions (e.g., ignoring and criticizing) invite similarly uncommunal reactions from others; conversely, agentic actions (e.g., asserting and leading) invite unagentic reactions (e.g., stepping back and yielding), whereas unagentic actions invite agentic reactions (e.g., stepping forward and taking charge). An intriguing implication is that communal or uncommunal behavior may evoke positive (amplifying) feedback loops that cause an entire group to become increasingly communal or increasingly uncommunal; conversely, agentic or unagentic behavior may evoke negative (dampening) feedback loops that prevent an entire group from becoming highly agentic or highly unagentic. If so, then the same complementarity dynamic that tends to polarize individuals within groups into very agentic and unagentic roles (e.g., with some members dominating decision-making and others rarely speaking) would simultaneously work against agentic or unagentic norms characterizing a group as a whole. This may explain why it was harder to identify reliable indicators of agentic/unagentic than communal/uncommunal cultures in Study 1, and why the agentic/unagentic [PA/HI] scales (compared to the communal/uncommunal [LM/DE] scales) tended to show weaker internal consistencies, weaker ICCs, and weaker correlations with other measures.

### Accuracy and Bias

The CCS/CTS—like any self-report measure—is vulnerable to response biases. Measuring group norms by aggregating multiple members’ CCS/CTS ratings reduces the influence of response biases that vary across individuals within groups (e.g., some group members making overly positive and other members making overly negative ratings) but can increase the influence of response biases that systematically vary across groups (e.g., some groups making overly positive and other groups making overly negative ratings).

As the preceding examples suggest, one common bias is evaluative bias, which reflects respondents considering not only an item’s descriptive substance but also its social desirability (Leising et al., 2015). Situations that foreground the evaluative implications of an assessment magnify evaluative bias. For example, because Study 3 participants knew that outside consultants would be conducting a feedback session immediately following the assessment, they may have been sensitive to whether their responses portrayed their team or organization positively or negatively, which may have contributed to their CCS/CTS responses not forming a perfect circumplex. On the other hand, even in that situation, the CCS/CTS met the criteria for a quasi-circumplex, which suggests that respondents were still principally responding to items’ descriptive content.

Of course, even when respondents honestly report their impressions of their organization or team, their impressions may be inaccurate. For example, to the degree that members of teams or organizations share simplified stereotypes of their group (e.g., “we’re go getters” or “we’re nurturers”), we may observe stronger within-group agreement and between-group differentiation than actually exists. In other words, the CCS/CTS measures a group’s *shared beliefs* about its interpersonal culture, and how accurately those beliefs reflect behavior is an empirical question that can only be answered by research that uses more objective indicators of behavior, such as observer ratings. For similar reasons, additional studies using more objective outcome measures (i.e., not just members’ own judgments) are necessary to more definitively determine which group cultures predict the best group outcomes.

### Future Directions

To enhance the utility of the CCS/CTS, it will also be important to go beyond simply identifying which circumplex profile is, on average, associated with better group outcomes. For example, future research using the CCS/CTS should examine whether the nature of the team (e.g., management versus non-management) or organization (e.g., financial services versus health services) moderates which interpersonal norms predict the best outcomes. Moreover, if interpersonal norms have consequences, then it is important to evaluate interventions to change norms, recognizing that the most efficient ways to change interpersonal norms may not be the most efficient ways to change other facets of organizational culture. For example, given how interpersonal norms spread, it may be most efficient to “seed change” through the behavior of a small number of respected employees and leaders who occupy highly visible or central nodes within the group’s social network (Valente, 2012).

As noted earlier, clinical researchers and practitioners who use multisurface interpersonal assessment (administering more than one IPC inventory) better understand specific clinical cases and diagnostic categories (e.g., Dawood and Pincus, 2016; Dowgwillo and Pincus, 2017). Multisurface assessment may prove useful to organizational researchers and practitioners as well. As a simple example, Study 3 groups completed the CCS/CTS twice—first assessing actual norms and then assessing ideal norms. Also administering the CLS would reveal segments of the IPC where leaders’ interpersonal styles do or do not align with their team’s or organization’s actual and ideal norms. Assessing other employees’ interpersonal styles and juxtaposing their circumplexes with the CCS/CTS circumplex may provide insight into their person-organization or person-group fit (Kristof-Brown et al., 2005) as well as the degree to which an employee’s behavior (e.g., not sharing information with co-workers) should be attributed to workplace norms or to the individual’s distinctive dispositions.

### Summary and Conclusion

Interpersonal circumplex inventories like the CCS/CTS offer several benefits. First, they systematically assess every segment of the space defined by the empirically supported, broadly applicable orthogonal factors of agency and communion. Second, they can parsimoniously summarize their findings as summary vectors or single points on the circumplex. Third, because all IPC instruments share the same model, their findings can be readily compared.

The CCS/CTS is unique among IPC inventories in assessing groups rather than individuals. Specifically, CCS/CTS ratings of organizations or teams—to the degree that they converge within groups and diverge between groups—express an organization’s or team’s distinctive collective understanding of its interpersonal culture. In the current research, the organizational and team cultures described as ideal and as yielding superior outcomes had very strong communal norms, moderately strong agentic norms, moderately weak unagentic norms, and very weak uncommunal norms.

While further validation is needed (e.g., using more objective criteria), the CCS/CTS scales demonstrated encouraging psychometric and circumplex properties in both online and onsite samples. By bridging circumplex and organizational literatures, the CCS/CTS is positioned to generate research that contributes to both areas. In sum, the CCS/CTS appears to offer a unique and useful tool for examining the social norms that comprise the heart of organizational and team culture.

## Ethics Statement

All subjects gave written informed consent in accordance with the Declaration of Helsinki. The research protocol was approved by the University of Idaho Institutional Review Board.

## Author Contributions

KL conceptualized and orchestrated the studies, analyzed the data, and wrote the manuscript.

## Conflict of Interest Statement

Although the CCS/CTS is available from the author (KL) free of charge for research or educational purposes, KL will receive a small royalty from the Culture Capital Group in connection with any commercial use.

## Footnotes

1 Almost all IPC inventories divide the IPC into the octants shown in Figure 1—namely, PA, BC, DE, FG, HI, JK, LM, and NO. An exception is the CLS (Redeker et al., 2014), which rotates the octant dividing lines by half an octant, yielding the following octant scales: AB, CD, EF, GH, IJ, KL, MN, and OP. Although the studies below only report psychometrics for the standard traditional octants (PA, BC, and so on), the Supplementary Materials include tables and figures displaying the corresponding psychometrics for the alternative octants (AB, CD, and so on).

2 https://osf.io/ygt5e

3 These participants also completed another brief organizational culture measure, but because its scales proved to be highly correlated (i.e., did not measure clearly distinguishable cultures), the results from this measure are reported in the Supplementary Materials.

4 As Figure 4 highlights, MCP Agreeableness and Extraversion are strongly positively correlated with highly communal–mildly agentic norms (the most satisfying norms) and negatively correlated with highly uncommunal–mildly unagentic norms (the least satisfying norms). Consequently, after controlling for the MCP, the additional variance in satisfaction explained was small. While the MCP is useful for quickly differentiating satisfied from dissatisfied groups, it has limitations. For example, as Figure 4 also highlights, the MCP is poor at differentiating highly agentic–mildly uncommunal (e.g., pushy) from highly unagentic–mildly communal (e.g., cautious) groups. Although these disparate work environments evoke similar (medium) levels of satisfaction, they presumably would be dissimilar on other measures of organizational processes and outcomes (e.g., frequency of innovations or ethical violations).

5 At this juncture, respondents completed 10 additional items measuring group processes or outcomes that consultants administering the materials believed would provide useful feedback to the participating groups (e.g., “I rarely think about looking for a job at another organization”). Because these ratings strongly correlated with the satisfaction ratings, and the items given to teams versus organizations were somewhat different, for simplicity, the results for these items are not reported.

6 Although the *Circumplex Scales of Intergroup Goals* concerns relationships between groups, its target is the *individual’s* goals for his/her group—e.g., how each respondent wants his/her organization to interact with other organizations (Locke, 2014)—rather than the goals of the group as a collective.
