# Supplementary material for: Development and Validation of a Circumplex Measure of the Interpersonal Culture in Work Teams and Organizations
Source: Front Psychol. 2019 May 1;10:850. doi: 10.3389/fpsyg.2019.00850 (PMC6504781; doi:10.3389/fpsyg.2019.00850)
Supplement: Supplementary file 1 [file Data_Sheet_1.PDF]

Supplementary Tables and Figures

Table S1

Descriptive Statistics and Intercorrelations for Organizational Culture Assessment Instrument (OCAI) and Satisfaction Scales – Study 2

				Correlations				
Variable	*M*	*SD*	α	1	2	3	4	5
1. OCAI Clan Culture	32.87	18.63	.75	—				
2. OCAI Adhocracy Culture	15.46	9.56	.81	-.16	—			
3. OCAI Market Culture	22.19	15.00	.71	-.70	.04	—		
4. OCAI Hierarchy Culture	29.47	15.13	.80	-.44	-.48	-.16	—	
5. Satisfaction	3.36	0.86	.70	.38	.06	-.41	-.10	—

Note. OCAI *n* = 544. Satisfaction *n* = 541 (i.e., only includes participants who also completed the OCAI). OCAI scores could range from 0 to 100; Satisfaction scores could range from 0 to 4. α = Cronbach's alpha. Correlations > .11 are significant at *p* < .01.

Table S2

Descriptive Statistics and Intercorrelations for Measure of Collective Personality (MCP) and Satisfaction Scales – Study 2

Variable	*M*	*SD*	α	1	2	3
1. MCP Agreeableness	3.01	.84	.94	—		
2. MCP Extraversion	2.73	.57	.79	.52	—	
3. Satisfaction	3.23	.94	.77	.54	.32	—

Note. MCP *n* = 869. Satisfaction *n* = 857 (i.e., only includes participants who also completed the OCAI). The response scale for all items ranged from 0 to 4. α = Cronbach's alpha. Correlations > .09 are significant at *p* < .01.

Table S3

Correlations and Summary Parameters for Relations between the CCS or CTS and the Organizational Culture Assessment Inventory (OCAI), Measure of Collective Personality (MCP), and Satisfaction Scales – Study 2

	Correlations with CCS/CTS Octant Scales								Summary Parameters				
Variables	PA	BC	DE	FG	HI	JK	LM	NO	Communal Vector *[CI]*	Agentic Vector *[CI]*	Angle *[CI]*	Amplitude *[CI]*	R^2
**OCAI**													
Clan													
CCS	.00	-.48	-.52	-.35	-.05	.39	.52	.40	.55 *[.46, .62]*	-.01 *[-.10, .08]*	358.9 *[349.1, 8.4]*	.55 *[.47, .63]*	0.99
CTS	-.09	-.41	-.40	-.26	-.13	.27	.47	.28	.43 *[.34, .52]*	-.01 *[-.10, .07]*	358.1 *[346.3, 9.3]*	.43 *[.34, .53]*	0.96
Adhocracy													
CCS	.35	.19	-.06	-.12	-.18	-.06	.09	.18	.05 *[-.08, .17]*	.23 *[.14, .32]*	78.8 *[49.6, 11.7]*	.23 *[.15, .33]*	0.94
CTS	.13	.15	-.06	-.07	-.05	-.02	.06	.07	.02 *[-.10, .14]*	.10 *[.02, .18]*	77.1 *[26.9, 149.3]*	.10 *[.04, .21]*	0.76
Market													
CCS	.02	.48	.52	.31	.05	-.36	-.50	-.36	-.52 *[-.61, -.42]*	.02 *[-.07, .12]*	177.3 *[166.7, 187.7]*	.52 *[.43, .62]*	0.99
CTS	.24	.49	.39	.19	-.02	-.31	-.44	-.14	-.41 *[-.52, -.29]*	.15 *[.06, .24]*	159.9 *[146.6, 171.2]*	.44 *[.32, .54]*	0.97
Hierarchy													
CCS	-.22	-.03	.14	.17	.11	-.07	-.17	-.22	-.16 *[-.27, -.04]*	-.15 *[-.25, -.04]*	223.3 *[195.3, 256.5]*	.21 *[.11, .33]*	0.98
CTS	-.20	-.05	.18	.19	.21	-.03	-.21	-.26	-.17 *[-.29, -.06]*	-.19 *[-.28, -.10]*	226.9 *[205.9, 250.7]*	.25 *[.16, .36]*	0.98
**MCP**													
Agreeableness													
CCS	.27	-.46	-.83	-.58	-.05	.57	.82	.61	.81 *[.77, .84]*	.11 *[.03, .18]*	7.7 *[2.3, 12.9]*	.81 *[.78, .85]*	0.99
CTS	.02	-.52	-.78	-.56	-.14	.50	.77	.50	.76 *[.71, .80]*	.05 *[-.02, .13]*	3.8 *[358.1, 9.5]*	.76 *[.71, .80]*	1.00
Extraversion													
CCS	.58	-.02	-.50	-.64	-.37	.14	.55	.64	.52 *[.46, .58]*	.44 *[.37, .50]*	40.1 *[33.7, 46.6]*	.68 *[.63, .73]*	0.99
CTS	.40	-.11	-.50	-.64	-.51	-.02	.44	.62	.47 *[.41, .54]*	.43 *[.38, .49]*	42.6 *[36.6, 49.0]*	.64 *[.59, .70]*	1.00
**Satisfaction**													

CCS	.24	-.18	-.45	-.39	-.12	.23	.50	.44	.46 *[.40, .51]*	.17 *[.11, .22]*	20.0 *[13.7, 26.4]*	.49 *[.43, .54]*	1.00
CTS	.12	-.24	-.43	-.38	-.15	.24	.49	.35	.45 *[.38, .51]*	.11 *[.06, .15]*	13.7 *[8.1, 19.6]*	.46 *[.40, .53]*	0.99

Note. *N*s = 789 for CCS x Satisfaction, 445 for CTS x MCP, and 807 for CTS x Satisfaction. PA = Courageous & Pushy, BC = Competitive & Combative, DE = Rude & Guarded, FG = Evasive & Hesitant, HI = Timid & Cautious, JK = Yielding & Modest, LM = Respectful & Open, NO = Engaged & Confident. Any correlations > .13 are significant at $p < .01$. R^2 = goodness-of-fit to ideal curve. *CI* = Confidence intervals computed using resampling procedures implemented by the circumplex package for R (Girard, Zimmerman, & Wright, 2018).

Table S4

CCS and CTS Scales' Descriptive Statistics, Intercorrelations, and Loadings on Communal and Agentic Principal Components – Study 3

	CCS		CTS		Correlations								Communal Loadings		Agentic Loadings	
Octant	*M*	*SD*	*M*	*SD*	PA	BC	DE	FG	HI	JK	LM	NO	CCS	CTS	CCS	CTS
PA	2.23	.20	2.25	.27		.30	-.17	-.56	-.62	-.40	.20	.59	.03	.05	.86	.86
BC	1.75	.25	1.56	.46	.27		.83	.45	.12	-.56	-.80	-.45	-.91	-.90	.22	.31
DE	1.26	.33	0.95	.58	-.14	.79		.79	.47	-.36	-.95	-.78	-.94	-.97	-.09	-.14
FG	1.52	.25	1.32	.40	-.44	.61	.73		.85	.09	-.75	-.93	-.76	-.72	-.57	-.65
HI	1.82	.26	1.68	.34	-.60	.31	.51	.85		.42	-.44	-.81	-.47	-.39	-.83	-.84
JK	2.19	.19	2.26	.28	-.33	-.63	-.53	-.07	.30		.42	-.12	.63	.50	-.63	-.71
LM	2.57	.34	2.70	.52	.19	-.78	-.91	-.78	-.53	.50		.77	.94	.97	.15	.12
NO	2.45	.27	2.60	.38	.56	-.54	-.71	-.89	-.82	.14	.79		.74	.72	.60	.66

Note. CCS n = 21; CTS n = 38. PA = Courageous & Pushy; BC = Competitive & Combative; DE = Rude & Guarded; FG = Evasive & Hesitant; HI = Timid & Cautious; JK = Yielding & Modest; LM = Respectful & Open; NO = Engaged & Confident. Ratings were on Strongly Disagree (0) to Strongly Agree (4) scales. In the correlation matrix, CCS scale intercorrelations appear below the diagonal and CTS scale intercorrelations appear above the diagonal. CCS correlations > .44 and CTS correlations > .32 are significant at $p < .05$. The factor loadings reflect Procrustean rotation aligning the first two principal components with the theoretical orientations of the communal and agentic dimensions.

Table S5

Actual and Ideal Social Norms for Organizations (CCS) and Teams (CTS) – Study 3

	CCS					CTS				
	Actual		*Ideal*			*Actual*		*Ideal*		
Octant	*M*	*SD*	*M*	*SD*	t(20)	*M*	*SD*	*M*	*SD*	t(36)
PA	2.23	*.21*	2.52	*.17*	-8.00**	2.26	*.28*	2.68	*.21*	-8.71**
BC	1.76	*.25*	1.44	*.20*	5.53**	1.57	*.49*	1.34	*.21*	2.66*
DE	1.32	*.38*	0.63	*.29*	8.60**	0.94	*.63*	0.29	*.21*	6.49**
FG	1.53	*.25*	0.97	*.24*	8.75**	1.31	*.38*	0.70	*.19*	11.84**
HI	1.81	*.27*	1.47	*.24*	5.57**	1.66	*.32*	1.26	*.21*	8.91**
JK	2.17	*.21*	2.19	*.29*	-.32**	2.25	*.30*	2.12	*.25*	2.24
LM	2.51	*.41*	3.19	*.28*	-8.38**	2.71	*.55*	3.48	*.20*	-8.65**
NO	2.43	*.30*	3.04	*.26*	-8.49**	2.62	*.39*	3.31	*.24*	-11.34**

Note. *N* = 21 organizations and 37 teams (one team did not rate ideal norms). * $p \leq .01$. ** $p \leq .001$.

Below are versions of Table 2, Table 3, Table 5, Table 7, Figure 2, and Figure 7 from the main text—plus versions of the above Supplementary Tables S4 and S5—using the alternative octants employed by the Circumplex Leadership Scan (CLS).

Table 2 Supplement – Alternative Octants

CCS Scales' Descriptive Statistics, Intercorrelations, and Loadings on Communal and Agentic Principal Components – Study 1

				Correlations								Loadings	
Octant	α	*M*	*SD*	AB	CD	EF	GH	IJ	KL	MN	OP	Communal	Agentic
AB	.83	3.28	.70	—								-.30	.70
CD	.91	2.52	.88	.53	—							-.86	.34
EF	.87	2.24	.71	.06	.61	—						-.83	-.35
GH	.77	2.47	.60	-.24	.14	.61	—					-.39	-.75
IJ	.81	2.88	.69	-.44	-.50	-.01	.44	—				.40	-.74
KL	.89	3.37	.80	-.35	-.78	-.54	-.04	.60	—			.87	-.30
MN	.89	3.74	.69	.04	-.55	-.74	-.44	.13	.66	—		.85	.32
OP	.77	3.54	.56	.44	-.02	-.50	-.62	-.30	.13	.61	—	.40	.78

Note. $n = 457$. AB = Competitive & Pushy; CD = Rude & Combative; EF = Evasive & Guarded; GH = Timid & Hesitant; IJ = Yielding & Cautious; KL = Respectful & Modest; MN = Engaged & Open; OP = Courageous & Confident. α = Cronbach's alpha. Ratings were on Strongly Disagree (0) to Strongly Agree (4) scales. The factor loadings reflect Procrustean rotation aligning the first two principal components with the theoretical orientations of the communal and agentic dimensions. Correlations > .12 are significant at $p < .01$.

Table 3 Supplement – Alternative Octants

CCS and CTS Octant Scale Descriptive Statistics, Reliabilities, Intercorrelations, and Loadings on Communal and Agentic Principal Components – Study 2

	CCS			CTS			Correlations								Communal Loading		Agentic Loading	
Octant	α	*M*	*SD*	α	*M*	*SD*	AB	CD	EF	GH	IJ	KL	MN	OP	CCS	CTS	CCS	CTS
AB	.83	2.18	.75	.82	2.09	.74		.57	.29	.09	-.16	-.27	-.04	.32	-.29	-.45	.69	.50
CD	.91	1.50	.96	.88	1.31	.86	.49		.71	.41	-.21	-.61	-.47	-.05	-.86	-.85	.27	.19
EF	.89	1.33	.84	.90	1.13	.84	.07	.66		.72	.11	-.48	-.63	-.35	-.82	-.84	-.35	-.34
GH	.78	1.65	.68	.79	1.52	.67	-.15	.28	.69		.40	-.11	-.40	-.40	-.46	-.52	-.70	-.66
IJ	.81	2.02	.72	.74	2.07	.67	-.37	-.42	-.02	.37		.53	.15	-.05	.43	.28	-.70	-.72
KL	.88	2.40	.84	.85	2.64	.74	-.26	-.74	-.56	-.15	.60		.66	.29	.87	.81	-.25	-.29
MN	.89	2.72	.79	.87	2.98	.68	.06	-.55	-.71	-.49	.18	.66		.61	.83	.79	.34	.27
OP	.81	2.54	.64	.76	2.70	.59	.36	-.13	-.46	-.52	-.08	.28	.66		.47	.39	.66	.62

Note. Circumplex Culture Scan (CCS) n = 808; Circumplex Team Scan (CTS) n = 832. AB = Competitive & Pushy; CD = Rude & Combative; EF = Evasive & Guarded; GH = Timid & Hesitant; IJ = Yielding & Cautious; KL = Respectful & Modest; MN = Engaged & Open; OP = Courageous & Confident. Ratings were on Strongly Disagree (0) to Strongly Agree (4) scales. α = Cronbach's alpha. In the correlation matrix, CCS scale intercorrelations appear below the diagonal and CTS scale intercorrelations appear above the diagonal. Correlations > .09 are significant at $p < .01$. The factor loadings reflect Procrustean rotation aligning the first two principal components with the theoretical orientations of the communal and agentic dimensions.

Table 5 Supplement – Alternative Octants

Within-Group Interrater Agreement (r_{wg}, a_{wg}) and Intraclass Correlation (ICC) Aggregation Indices

Target / Scale	$r_{WG(J).uniform}$	$r_{WG(J).normal}$	$a_{WG(J)}$	ICC(2)	ICC(1)	F ratio
Organizations						
AB	.94	.80	.68	.75	.11	3.98**
CD	.90	.43	.52	.72	.10	3.61**
EF	.93	.49	.60	.80	.14	5.03**
GH	.93	.71	.63	.74	.10	3.79**
IJ	.92	.70	.63	.54	.05	2.16**
KL	.92	.65	.61	.76	.11	4.11**
MN	.92	.67	.61	.83	.17	5.95**
OP	.94	.80	.68	.74	.10	3.88**
Teams						
AB	.92	.67	.64	.59	.14	2.44**
CD	.92	.68	.58	.85	.38	6.60**
EF	.94	.76	.63	.86	.40	7.08**
GH	.93	.73	.67	.79	.29	4.79**
IJ	.91	.58	.62	.63	.16	2.74**
KL	.92	.64	.62	.74	.24	3.85**
MN	.93	.67	.64	.84	.37	6.34**
OP	.94	.80	.71	.78	.28	4.56**

Note. N = 347 CTS respondents from 38 teams, and 516 CCS respondents from 21 organizations. AB = Competitive & Pushy; CD = Rude & Combative; EF = Evasive & Guarded; GH = Timid & Hesitant; IJ = Yielding & Cautious; KL = Respectful & Modest; MN = Engaged & Open; OP = Courageous & Confident. *SD* = standard deviation of r_{WG} values. ** $p < .005$.

Table 7 Supplement – Alternative Octants

Paired Samples t-tests of Actual and Ideal Social Norms – Study 3

	Actual		Ideal		
Octant	*M*	*SD*	*M*	*SD*	t(57)
AB	1.90	.30	1.97	.21	-1.82
CD	1.30	.53	0.79	.25	7.73**
EF	1.21	.45	0.54	.29	12.35**
GH	1.57	.33	1.06	.22	13.18**
IJ	1.89	.28	1.64	.31	6.09**
KL	2.52	.40	2.92	.22	-7.22**
MN	2.63	.50	3.40	.29	-12.39**
OP	2.44	.30	2.96	.26	-13.49**

Note. N = 58 teams and organizations (one team did not rate ideal norms). ** $p < .001$

Supplementary Table S4 Supplement – Alternative Octants

CCS and CTS Scales' Descriptive Statistics, Intercorrelations, and Loadings on Communal and Agentic Principal Components – Study 3

	CCS		CTS		Correlations								Communal Loadings		Agentic Loadings	
Octant	*M*	*SD*	*M*	*SD*	AB	CD	EF	GH	IJ	KL	MN	OP	CCS	CTS	CCS	CTS
AB	2.00	.23	1.85	.31		.67	.48	.03	-.13	-.56	-.38	.13	-.64	.33	-.68	.48
CD	1.46	.30	1.18	.53	.58		.89	.46	.10	-.90	-.82	-.35	-.96	.11	-.97	.09
EF	1.37	.29	1.12	.49	.41	.86		.73	.40	-.80	-.95	-.65	-.91	-.33	-.94	-.28
GH	1.68	.26	1.52	.36	.16	.54	.80		.75	-.38	-.73	-.89	-.62	-.74	-.56	-.78
IJ	1.96	.20	1.87	.32	-.03	.00	.36	.71		.07	-.42	-.73	-.09	-.90	-.17	-.88
KL	2.42	.27	2.61	.39	-.56	-.93	-.78	-.42	.15		.75	.28	.94	-.20	.92	-.18
MN	2.56	.33	2.70	.50	-.32	-.77	-.92	-.81	-.36	.74		.67	-.64	.33	-.68	.48
OP	2.34	.24	2.47	.33	-.07	-.35	-.70	-.84	-.63	.37	.74		-.96	.11	-.97	.09

Note. CCS $n = 21$; CTS $n = 38$. AB = Competitive & Pushy; CD = Rude & Combative; EF = Evasive & Guarded; GH = Timid & Hesitant; IJ = Yielding & Cautious; KL = Respectful & Modest; MN = Engaged & Open; OP = Courageous & Confident. Ratings were on Strongly Disagree (0) to Strongly Agree (4) scales. In the correlation matrix, CCS scale intercorrelations appear below the diagonal and CTS scale intercorrelations appear above the diagonal. CCS correlations > .44 and CTS correlations > .32 are significant at $p < .05$. The factor loadings reflect Procrustean rotation aligning the first two principal components with the theoretical orientations of the communal and agentic dimensions.

Supplementary Table S5 Supplement – Alternative Octants

Actual and Ideal Social Norms for Organizations (CCS) and Teams (CTS) – Study 3

	CCS					CTS				
	Actual		*Ideal*			*Actual*		*Ideal*		
Octant	*M*	*SD*	*M*	*SD*	t(20)	*M*	*SD*	*M*	*SD*	t(36)
AB	2.00	*.24*	1.99	*.17*	.10	1.85	*.32*	1.96	*.23*	-2.12
CD	1.50	*.33*	.93	*.26*	8.87**	1.19	*.60*	.71	*.21*	4.92**
EF	1.40	*.30*	.76	*.25*	9.56**	1.11	*.48*	.42	*.23*	8.95**
GH	1.69	*.26*	1.20	*.23*	7.34**	1.50	*.35*	.98	*.18*	10.87**
IJ	1.95	*.21*	1.77	*.31*	2.85*	1.86	*.31*	1.57	*.29*	5.47**
KL	2.38	*.32*	2.82	*.17*	-6.45**	2.60	*.42*	2.97	*.22*	-4.80**
MN	2.51	*.39*	3.22	*.30*	-8.70**	2.70	*.55*	3.50	*.23*	-9.37**
OP	2.34	*.23*	2.78	*.22*	-7.25**	2.49	*.32*	3.07	*.23*	-11.62**

Note. N = 21 organizations and 37 teams (one team did not rate ideal norms). * $p \leq .01$. ** $p \leq .001$.

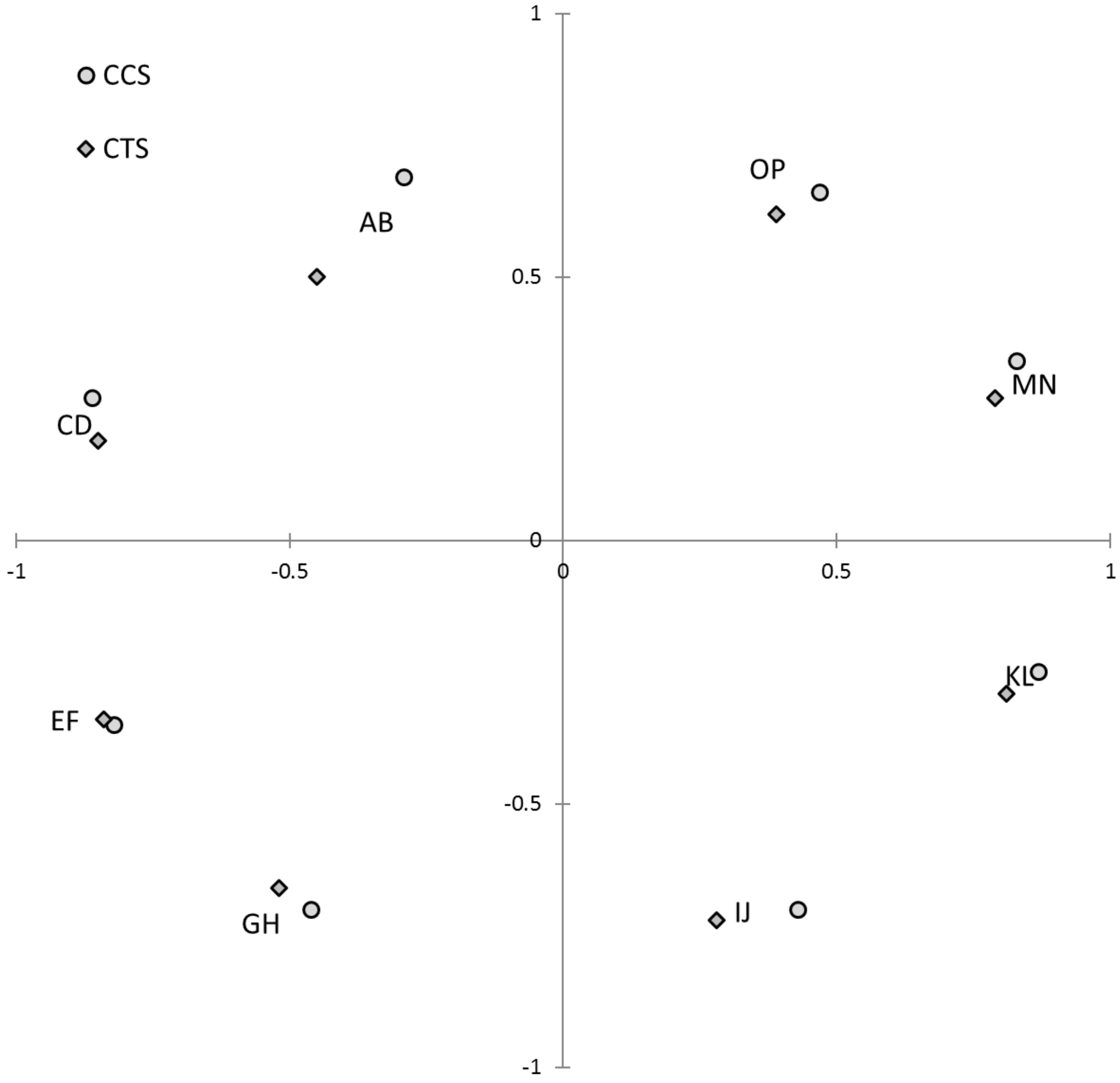

Figure 2 Supplement – Alternative Octants. Structure of the Circumplex Culture Scan (CCS) and Circumplex Team Scan (CTS) scales (Study 2). Solution rotated for maximum convergence to theoretical angular locations. AB = Competitive & Pushy; CD = Rude & Combative; EF = Evasive & Guarded; GH = Timid & Hesitant; IJ = Yielding & Cautious; KL = Respectful & Modest; MN = Engaged & Open; OP = Courageous & Confident.

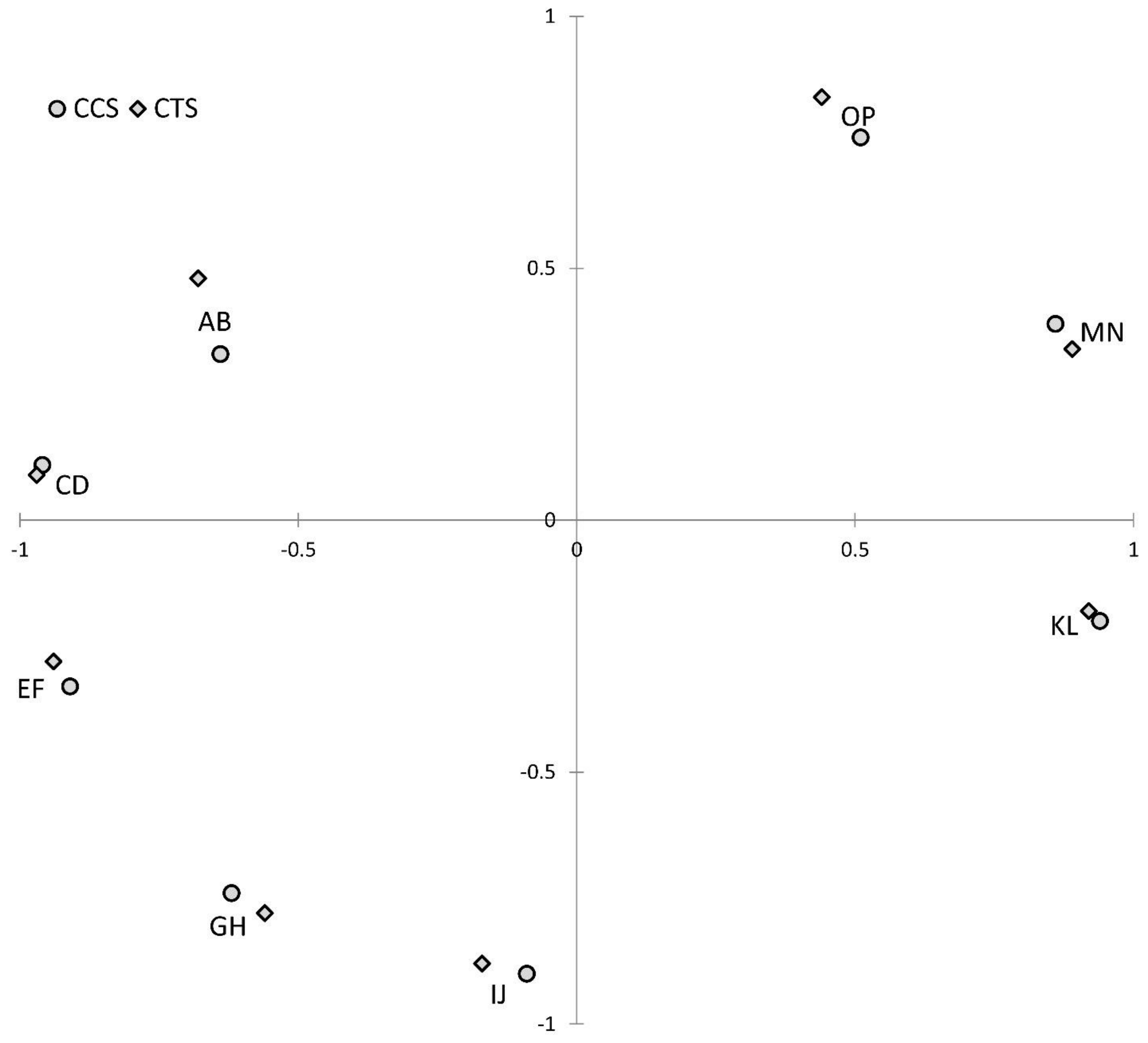

Figure 7 Supplement – Alternative Octants. Structure of the Circumplex Culture Scan (CCS) and Circumplex Team Scan (CTS) scales (Study 3). Solution rotated for maximum convergence to theoretical angular locations. AB = Competitive & Pushy; CD = Rude & Combative; EF = Evasive & Guarded; GH = Timid & Hesitant; IJ = Yielding & Cautious; KL = Respectful & Modest; MN = Engaged & Open; OP = Courageous & Confident.
